# A Data-Independent Methodology for the Structural Characterization of Microcystins and Anabaenopeptins Leading to the Identification of Four New Congeners

**DOI:** 10.3390/toxins11110619

**Published:** 2019-10-26

**Authors:** Audrey Roy-Lachapelle, Morgan Solliec, Sébastien Sauvé, Christian Gagnon

**Affiliations:** 1 Environment and Climate Change Canada, Aquatic Contaminants Research Division, Montréal, QC H2Y 2E5, Canada; audrey.roy-lachapelle@canada.ca; 2 NSERC-Industrial Chair on Drinking Water, CGM Department, École Polytechnique de Montréal, Montréal, QC H3T 1J4, Canada; morgan.solliec@polymtl.ca; 3 Department of Chemistry, Université de Montréal, Montréal, QC H3T 1J4, Canada; sebastien.sauve@umontreal.ca

**Keywords:** cyanotoxins, cyanobacteria, microcystins, anabaenopeptins, LC-HRMS, data-independent acquisition, suspect screening, non-target screening, structural characterization

## Abstract

Toxin-producing cyanobacteria are responsible for the presence of hundreds of bioactive compounds in aquatic environments undergoing increasing eutrophication. The identification of cyanotoxins is still emerging, due to the great diversity of potential congeners, yet high-resolution mass spectrometry (HRMS) has the potential to deepen this knowledge in aquatic environments. In this study, high-throughput and sensitive on-line solid-phase extraction ultra-high performance liquid chromatography (SPE-UHPLC) coupled to HRMS was applied to a data-independent acquisition (DIA) workflow for the suspect screening of cyanopeptides, including microcystin and anabaenopeptin toxin classes. The unambiguous characterization of 11 uncommon cyanopeptides was possible using a characterization workflow through extensive analysis of fragmentation patterns. This method also allowed the characterization of four unknown cyanotoxins ([Leu^1^, Ser^7^] MC-HtyR, [Asp^3^]MC-RHar, AP731, and AP803). The quantification of 17 common cyanotoxins along with the semi-quantification of the characterized uncommon cyanopeptides resulted with the identification of 23 different cyanotoxins in 12 lakes in Canada, United Kingdom and France. The concentrations of the compounds varied between 39 and 41,000 ng L^−1^. To our knowledge, this is the first DIA method applied for the suspect screening of two families of cyanopeptides simultaneously. Moreover, this study shows the great diversity of cyanotoxins in lake water cyanobacterial blooms, a growing concern in aquatic systems.

## 1. Introduction

Eutrophication of natural water sources is closely linked to the distinctive appearance of massive and episodic proliferations of cyanobacteria. These prokaryotic organisms do not systematically carry the expressed genes for toxin production, yet about 40 of the 150 cyanobacteria genera do possess these genes [1]. For more than two decades, microcystins (MCs) have been the main family of cyanopeptides extensively studied. This dominance has been triggered by tragic incidents, such as in a Brazilian hospital in 1996, where 52 patients undergoing dialysis succumbed to liver failure caused by contaminated water with MCs [2]. Following this, the World Health Organization (WHO) has suggested regulation levels for MC-LR in drinking water (1 µg L^−1^) which was extended by the US EPA to MC-LR equivalents to include more congeners and other cyanotoxins [3,4]. However, several families of cyanopeptides have long been identified along with MCs isolated from common cyanobacteria, i.e., *Microcystis* sp. Amongst them, cyanopeptolins, anabaenopeptins (APs), aerucyclamides, aeruginosines, and microginins are to mention when specifying the dominant families [5]. Still, the high diversity of produced congeners from each family and the little information known about factors and mechanisms linked to their production greatly complicates their study.

Potential cyanopeptides toxicity critically depends on the variants structure, but is still misunderstood and poorly documented [5]. MCs are hepatotoxic and readily accumulated in the liver from the specific binding to protein phosphatases 1 and 2A. The latter causes disruption of cellular homeostasis, and, in most acute cases, leads to liver necrosis, as well as colorectal and liver cancer [6]. Thus far, bioactive APs are considered non-toxic. Nevertheless, a few studies suggest that some APs congeners, such as AP-A, may demonstrate the potential to inhibit protease and protein phosphatases [7]. Moreover, AP-B and -F induce cyanobacteria lysis, ultimately affecting the bioavailability of other cell-bound cyanotoxins [8]. Accordingly, much still needs to be done on the unambiguous identification of these cyanopeptides and the assessment of their potential toxicity.

**Figure 1:**
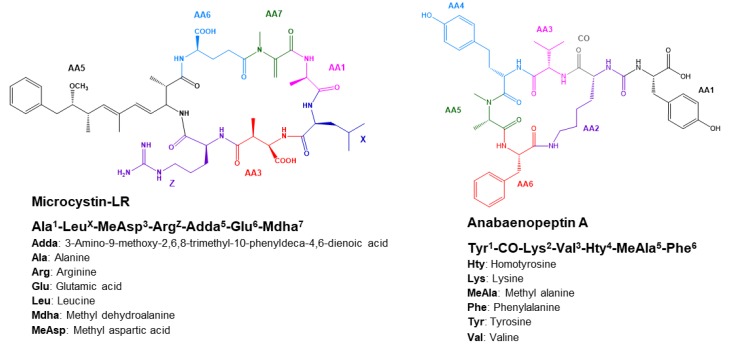
Cyclic non-ribosomal peptides structure of cyanopeptides MC-LR and AP-A.

Cyanopeptide’s structures are characterized by cyclic or linear non-ribosomal peptides, each family possessing a characteristic substructure and some variable amino acids and peptides. These variations in the core structure of each cyanopeptide multiply the number of combinations which is the cause of the large variety of potential congeners; to date, more than 500 cyanopeptides, including 240 MCs and 96 APs have been identified [7,9,10]. More specifically, MCs are cyclic heptapeptides (Figure 1) with a characteristic β-amino acid moiety named Adda (3-amino-9-methoxy-2,6,8-trimethyl-10-phenyldeca-4,6-dienoic acid), and two distinctive positions with the highest variation of monomers (X and Z). APs are cyclic peptides bound through a characteristic ureido-linkage (Figure 1); their structure is characterized as the following: AA1-CO-[Lys-AA3-AA4-MeAA5-AA6] with AA representing a variable amino acid residues and brackets, including the cyclic structure [9]. Based on the various amino acid combinations identified for these two families, an extensive list of potential amino acids per variable sites can be proposed to enumerate all possible theoretical combinations of cyanopeptides identifiable to date [11]. Based on the proposed combinations, one could theoretically propose a significantly higher number of congeners, although most of the variants may not occur naturally in practice, due to the low frequency of some amino acids in the possible combinations.

High-resolution mass spectrometry (HRMS) can use exact mass measurement coupled to database and software packages to become an increasingly more effective tool regarding the accurate identification of the suspect and unknown compounds without the use of certified standards, where target analysis is unfeasible. Suspect and non-target screening are the two main strategies used for the exhaustive search of the known and unknown compound where almost no reference material is available. In recent years, the use of these screening techniques in the environmental field has greatly increased, particularly for the non-target analysis of pharmaceuticals, pesticides, hormones in surface and treated water [12,13]. Reversewise, the presence of cyanotoxins in surface water has only been investigated by few authors using this type of analysis [11,14,15,16]. Isobaric interferences and co-eluting substances can represent major challenges in the identification process of a compound even when using HRMS. Moreover, a sole analysis, based on the accurate mass, is insufficient to confirm a structural identification, e.g., determining the degradation by-products or metabolites related to a compound of interest. A non-target screening method should include various confirmatory elements, such as the accurate mass (*m/z*), mass defect, isotopic pattern, charge states, adducts and fragmentation pattern that increase the confidence of identification [17]. Suspect screening includes the benefit and disadvantage to depend on suspect lists. It is mainly based on some of the information mentioned above for the identification, but a major drawback comes from a lack of data in online libraries for some small molecule families, i.e., cyanotoxins, which allow a formal identification [17]. Nonetheless, considering the possibility to build specific in-house databases for the unambiguous identification of the known and unknown cyanopeptides is promising for the study and identification of less known congeners. 

Several analytical strategies have been employed in the past to identify new cyanopeptide structures. Historically, nuclear magnetic resonance (NMR) was the method of choice regarding the structure elucidation of new cyanopeptides, sometimes combined with mass spectrometry (MS), but has been mainly applied only on cyanobacterial cultures and blooms where the cyanotoxins are typically found at higher concentrations and the matrices are less complex [18,19,20,21]. In environmental samples, the toxins are not concentrated enough for this technique. Therefore, MS-based methods with unambiguous identification are widely used. Matrix-assisted Laser Desorption/Ionization Time-of-Flight (MALDI-TOF) systems are used for accurate and simultaneous identification and quantification analysis in many complex matrices [7,22,23,24,25]. For higher sensitivity and selectivity, liquid chromatography (LC) coupled to HRMS are increasingly used for quantitative and qualitative analysis. In the past, the LC coupled to several MS analyzers has proven effective in identifying unknown cyanopeptide congeners: Tandem mass spectrometry [26,27], Q-Trap [7,28], Q-TOF [11,15,29], Orbitrap^TM^ [14,30,31], and FTICR [32]. The fragmentation of precursor ions is key to allow unambiguous identification through the different amino acids and peptides, which are identifiable via their specific fragmentation spectra. Very few studies were developed to propose suspect screening methods for the analysis of MCs and APs in freshwater samples, whereas, most strategies are based on non-target screening of which the use of databases may not be necessary [11,15].

Most studies use data-dependent acquisition (DDA) to generate a wide full scan (FS) and fragmentation (MS/MS) information, which selects precursor masses with a list of exact masses that trigger the fragmentation of the most intense precursors, i.e., top 10 [33]. DDA is a very specific and useful method for non-target screening, but lacks speed when the suspect lists are too large to manage and could be limited by the duty cycle of the instrument, leading to MS/MS data loss of the less intense precursors. Data-independent acquisition (DIA) is another type of experiment that induces fragmentation of all precursor ions in a selected *m/z* window. DIA is very useful when fragmentation patterns of suspect compounds are known, but the MS/MS data generated by this acquisition mode are highly complex and may be difficult to interpret, due to co-eluting compounds from the sample or undesirable compounds from the matrix [33]. 

In this respect, a suspect screening strategy, based on a DIA experiment, was developed along with a generated list of candidates for the unambiguous identification of uncommon MCs and APs congeners. The study mainly focused on MCs and APs, which were the two main groups of compounds found in the sampling regions (southern Quebec lakes, Canada) [34]. A DIA-based method was developed with an automated solid phase extraction coupled to ultra-high liquid chromatography with heated electrospray ionization, and detection by a Q-Orbitrap^TM^ (SPE-UHPLC-HRMS) [34]. This integrated strategy allowed a sensitive and high-throughput analysis with minimum sample treatment for unambiguous identification of MCs and APs in freshwater. Two in-house databases with theoretical masses were built, including 660,960 MCs and 61,152 APs based on the combination of all the experimental peptides found to date in each compound family. To report the evidence of uncommon cyanopeptides, an optimization of analytical protocols was then described, and an identification strategy, based on levels of confidence, was applied [17,33,35]. A thorough discussion explained the optimized workflow, which led to the identification and characterization of MCs and APs, some of which have yet been unreported in the literature. Ultimately, a quantification of common cyanotoxins was done in real field samples, and a semi-quantification of suspected MCs and APs was achieved without the use of certified standards to estimate their environmental concentration. To our knowledge, this is the first report of a suspect screening strategy for the identification of known and unknown MCs and APs simultaneously along with the characterization of new cyanopeptides using a DIA-based method.

## 2. Results and Discussion

### 2.1. HRMS Parameters for Suspect Screening via DIA

The optimization of UHPLC-HRMS parameters for the suspect screening was focused on the MCs and APs analysis. The choice of these two families is the result of their high frequency in toxic algal blooms in selected freshwater sampling sites [34]. The FS mode acquisition window was chosen to include [M+H]^+^ and [M+2H]^2+^ ions, being set between *m/*z 300 and *m/z* 1400. This mass range includes relatively low masses which results in a more complex mass spectrum, due to the presence of matrix related compounds [11]. Thus, the use of Compound Discoverer 3.0 software (CD) (Thermo Fisher Scientific, San Jose, CA, USA) for raw data treatment has helped to significantly reduce the complexity of the dataset, by using a database and by targeting adducts. The establishment of appropriate chromatographic and mass spectrometry settings also allowed the reduction of the complexity of mass spectrum data.

Optimal fragmentation energy from the higher-energy C-trap dissociation (HCD) cell is needed to ensure an appropriate fragmentation of each compound. This appropriate fragmentation allows obtaining the best signal-to-noise (S/N) ratio for characteristic fragments ions, while keeping a small intensity, i.e., 10% of the parent ion in the FS spectrum. Both parent and product ions are used for the identification step. MCs and APs have similar structures, but the needed energy values for fragmentation differ depending on the combinations of amino acids in the basic structure and the ion form. Collision energies were manually optimized for MCs and APs individually by directly injecting certified standards in the ion source. As a result, stepped normalized collision energies (NCE) of 10, 20 and 30 were applied to induce the fragmentation of all suspect compounds optimally. 

**Figure 2:**
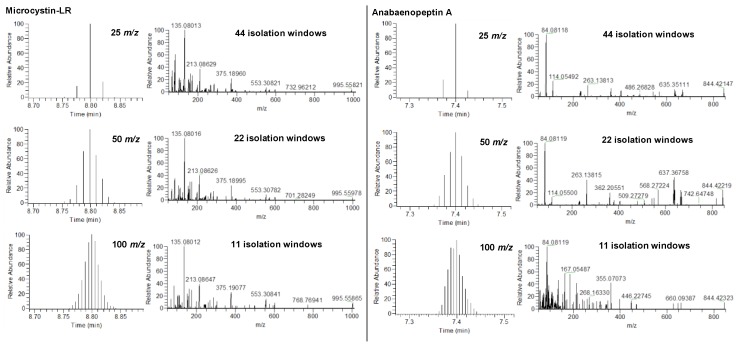
Optimization of the number of isolation windows for the DIA experiment for MCs and APs according to the *m/z* width and the number of isolation windows.

The DIA mode has been explained in numerous studies and results in complex mass spectra [17,33,36,37,38]. Several consecutive mass isolation windows (selected by the quadrupole) were included in the DIA strategy. Indeed, these mass isolation windows are subjected to an all ion fragmentation (AIF) scan mode which fragments all precursor ions from the whole mass dynamic range which explains the complexity of the fragmentation spectra after acquisition [17,36]. These isolation mass ranges have been voluntarily narrowed compared to what we can observe in the literature to reduce the complexity of the acquired data. By fragmenting these narrowed isolation windows, one can, therefore, significantly reduce the amount of data per spectra—thus, facilitating the interpretation of data and compound identification [36]. With this in mind, the number of isolation windows should be as high as possible to simplify the raw data, but the dual time may not be sufficient to obtain enough acquisition points per chromatographic peaks and adequate analytical results [38]. An optimization of the number of isolation windows is presented in Figure 2 for MC-LR and AP-A, and three *m/z* width values were tested, i.e., 25 *m/z*, 50 *m/z* and 100 *m/z* resulting in, respectively, 44, 22 and 11 isolation windows (on a *m/z* 300–1400 FS acquisition window). From 22 to a higher number of isolations windows, which means with 25 *m/z* width, the fragmentation spectra quality was enhanced. However, the dual time of the mass spectrometer was not sufficient to acquire enough points per peaks (i.e., around 10) for a quality identification analysis. On the other hand, identification was made easier with a higher number of isolation windows, thus 22 and higher. Best results were finally obtained with isolations windows of 50 *m/z* width, which gave the best compromise between the fragmentation spectra quality and the number of acquisition points per peak [38].

### 2.2. Building in-House Databases

To build in-house databases, which include all theoretical MCs and APs exact masses, exhaustive lists of the amino acids on each site of the peptide chains of these two compounds were built from several sources of literature, based on the structure of all known MCs and APs [5,7,9,10,11,28]. These lists are presented in Supplementary Information Figure S1. A macro was later built on an Excel^®^ file to generate in silico the adequate amino acid combinations from each list. Respectively 660,960 and 61,152 exact theoretical masses for MCs and APs were then obtained from this calculation. For each combination, the amino acid exact masses were summed to obtain the monoisotopic molecular weights of each theoretical compound. Then, this in silico generated lists of exact masses were included as a database for the raw data processing by CD software. However, these lists are massive, and include a large number of duplicate values. Thus, to facilitate automated raw data processing, the lists were reduced to suppress all duplicates, and these included 8709 and 8815 unique exact masses for MCs and APs, respectively. Finally, an Excel^®^ file was built from the first lists, which included the detailed combinations for each theoretical compound. These combination lists could then be used to identify potential new combinations of amino acid, and thus, new cyanopeptides and be validated by the manual interpretation of MS/MS spectra, which is explained in Section 2.4.

### 2.3. First Features Selection with Compound Discoverer

Using the previously described workflow through CD software, different lists of features were generated. The FS acquisition data included in the DIA experiment was set for the data treatment and features search. Aside the in-house lists, including all theoretical MCs and APs exact masses that were correlated with the FS data in this study, a search of features in other available online database (ChemSpider and mzCloud^TM^) was also included in the data treatment workflow to look at the total number of features that can be identified in the selected environmental samples. Overall, after blank subtraction, the number of selected features by CD varied between about 1600 and 6000 in the twelve lakes selected for this study (see Table S1 for more details on the samples). These numbers were consistent with most untargeted studies and can be tedious to interpret in order to find features relevant to one’s research [39,40,41]. This is where the selection criteria were useful to narrow down these lists, while searching for new cyanopeptides. First, the use of exact masses compared to the built-in database reduced the number of features of 12 to 116 for MCs and APs that would be identified as level 5 compounds (exact mass only) according to the identification levels strategy and confidence proposed by several studies [17,33], (Table S2). From there, the lists were narrowed down by selecting features with an appropriate isotopic pattern, adducts, retention times (RT), molecular formula and appropriate standard deviation (SD), resulting to lists of 3 to 51 features identified with level 3 confidence (tentative candidates by chemical class) [33]. Going further, distinctive fragments were used to strengthen the identification, and as described in Section 4.4, these fragments are common to all congeners of MCs and APs and were searched in the MS/MS spectra of the DIA experiment. After the feature’s selection, the lists were finally reduced between 0 and 17 features depending on compounds and samples. These features would then be taken to the last level of identification, which is a further manual study of the MS/MS spectra in order to make a structural identification of the features to finally identify them as potential or confirmed compounds. In short, the first lists of potential features were reduced, a 10-fold. Although this first selection was made automatically by CD, apart from the search for specific fragments, this exercise showed the importance of using rigorous criteria for unambiguous identification of features needed for the confirmation of the structure. Those would lead to a level 2 (probable structure by spectrum match) or a level 1 (confirmed structure by reference standard) characterization [17,33].

### 2.4. Confirmation of Suspects Using MS/MS Spectra

For an exhaustive identification of suspect compounds and to confirm their identify using fragmentation patterns, samples were re-analyzed using a parallel reaction monitoring (PRM) scan mode with inclusion lists, including the last selected features of MCs and APs for each sample set at the same retention time. Using PRM scan mode enabled to generate quality MS/MS spectra that would be easier to interpret with more specific fragmentation spectra. In parallel, a theoretical list of fragments was built in accordance with the literature for MCs and APs, including the most encountered amino acids combinations found in fragmentation spectra to better interpret the often-complex spectra [7,26,29,32,42]. The identification workflow presented below is based on characteristic fragments of unique amino acids or the addition of multiple amino acids found in MS/MS spectra. The strategy is to narrow down the number of candidates by identifying amino acids one by one in the structure until the structure can be confirmed.

#### 2.4.1. Microcystins Structures Elucidation

Before identifying unknown MC candidates, the workflow was tested with the elucidation of MC-LR found in sample no. 3 and confirmed with a certified standard shown in Table 1 and Figure S2. This example confirmed the accuracy of the list of specific fragment ions compared to the mass spectra. The structure elucidation workflow is presented in the next paragraph with the first example, *m/z* 1105.59150 found in sample no. 12. In this case, 482 different combinations of MCs within the 5 ppm mass accuracy range, where found with this exact mass (Table S3). In other words, this exact mass corresponds to a large number of combinations identified at level 5 of characterization. This is why it is necessary to add an MS/MS interpretation to confirm the compound structure.

**Figure 3:**
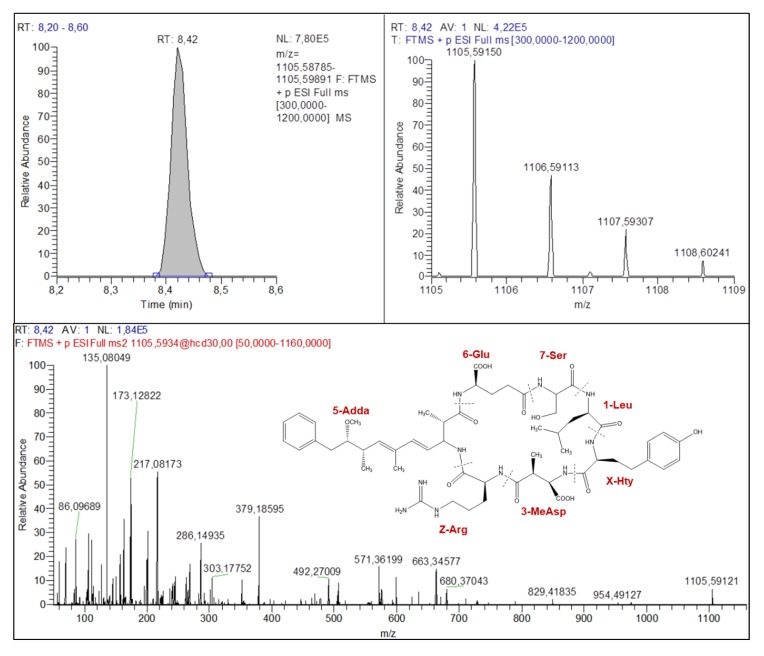
Chromatogram, isotopic pattern and fragmentation spectra of feature *m/z* 1105.5915 identified as [Leu^1^, Ser^7^]MC-HtyR with RT at 8.42 min.

For the structure elucidation workflow, a fragment ion was first used to confirm the presence of any form of the Adda moiety. This fragment ion would be present in all MCs’ structures and corresponds to *m/z* 163.11229. Afterwards, a second specific fragment ion was used to identify the form of Adda moiety found in the compound according to the following masses: *m/z* 135.08099 for Adda and (*6Z*)Adda, *m/z* 121.06534 for DMAdda and *m/z* 163.07591 for ADMAdda). In the case of *m/z* 1105.59150, specific fragment ions *m/z* 163.11150 and 135.08049 were identified in the fragmentation spectra of sample no. 12 (Figure 3 and Table 1 for fragment ions identification details). These fragment ions demonstrate the presence of the group Adda or (*6Z*)Adda, an isomerization form with weaker biological activity [43]. Subsequently, the amino acid in position 6 (AA6) can be identified by the fragment ion characterized by [Adda-134+AA6-NH_3_+H]^+^ (Table 1). Its exact mass *m/z* 292.15414 was found leading to the amino acid Glu. A third amino acid, Z, is then identifiable using the peptide ions [Z+Adda+AA6-CO+H]^+^ and [Z+Adda+AA6+H]^+^ with respective exact masses *m/z* 571.36199 and 599.36213. These exact masses are associated with the amino acid Arg. With these amino acids identified, AA3 is available to elucidation using peptide ions [AA3+Z-NH_2_+H]^+^, [AA3+Z+Adda+H]^+^ and [Z+Adda+AA6+AA3+H]^+^ fragments which were found to be *m/z* 286.14935, 599.36213 and 728.39614. These fragments were associated with MeAsp. Amino acid X was identified using its immonium ion, [X+AA3+H]^+^ and [X+AA3+Z+H]^+^ which were found, respectively, at *m/z* 129.11358, 307.12806 and 463.22949. These fragment ions are associated with Hty. At this point, AA1 and AA7 are easily identified using the list of potential fragment ions (Table 1), and these two last amino acids were identified as Leu and Ser, respectively. The last step of data mining in the fragmentation spectra is finally done to identify a maximum of different fragment ions to strengthen structure characterization. Finally, for this feature, two different compounds were identified, due to the potential presence of Adda or (*6Z*)Adda and the level of identification is set at level 2 since a certified standard would have confirmed the identification of the compound. We have identified this compound as [Leu^1^, Ser^7^]MC-HtyR, and this is to our knowledge the first time this compound was identified [10]. For each step of amino acids identification, a number of potential amino acids combinations were listed and are shown in Table S3. It shows that when only Adda was identified, 300 potential MCs were associated with the exact mass of this MC and when all the amino acids are identified, what are the potential MCs associated with the exact mass. This identification process was applied to the different features that were confirmed to be MCs, which is described in Table 1. The other features were identified as MCs, and corresponded to *m/z* 1009.57104, 1071.55340, 1085.56928 and 1038.57291. Each of these features were associated with six different potential compounds, due to two amino acid sites (Adda or (*6Z*)Adda and Mdha, Dhb or (*Z*)Dhb at position AA7). Considering the abundance of each amino acid, the compounds were identified as [GluOMe^6^]MC-LR in sample no. 3 (Figure S3), [M(O)^1^]MC-LR in sample no. 3 (Figure S4), [M(O)^1^, GluOMe^6^]MC-LR (Figure S5) in sample no. 3 and [Asp^3^]MC-RHar in sample no. 9 (Figure S6). The three firsts were already identified in previous studies, and the last is also an unknown cyanotoxin [32]. However, to confirm the identification of these MCs, certified standards would be needed. 

#### 2.4.2. Anabaenopeptins Structures Elucidation

A second identification workflow to identify APs was tested for the elucidation of AP-A found in sample no. 5 and confirmed with a certified standard, shown in Table 2 and Figure S7. This demonstrates the list of specific fragment ions associated with the mass spectra. The identification workflow for the structure elucidation of APs found in the lake water samples is detailed in the next paragraph.

**Figure 4:**
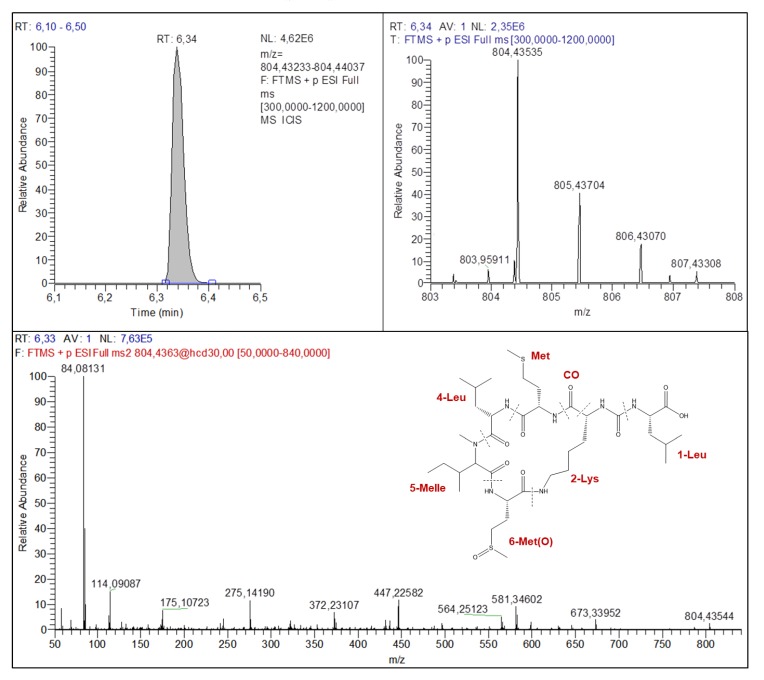
Chromatogram, isotopic pattern and fragmentation spectra of feature *m/z* 804.43535 identified as AP803 with RT at 6.34 min.

To identify potential APs, the first fragment ion used to narrow down the feature list is *m/z* 84.08136, an immonium fragment ion of lysine. Using this fragment ion alone, the features lists were reduced significantly, with 0 to 8 possible candidates identified with exact masses alone (Table S4). For the feature found at *m/z* 804.43535, only 11 potential combinations were found in the APs list (Table S4). Two fragment ions, [M+H-AA1-H_2_O]^+^ and [M+H-CO-AA1-H_2_O]^+^ (*m/z* 673.33952 and 645.34572), were used to identify AA1 (Figure 4 and Table 2) which was found to be Leu or Ile. With the list lowered at eight possible combinations, the theoretical fragment ion list was directly used to identify all the other amino acids and a new AP identified as AP803 with a structure described as (Ile or Leu)^1^-CO-Lys^2^-Met^3^-Leu^4^-MeIle^5^-Met(O)^6^ according to fragmentation spectra (Figure 4 and Table 2). Another new AP was identified at *m/z* 732.39224 according to fragmentation spectra (Figure S8 and Table 2), found in sample no. 11. In this case, this AP731 was the only candidate in the combination list, which leads to one structure elucidated to be Phe^1^-CO-Lys^2^-Val^3^-Leu^4^-MeGly^5^-AcSer^6^. Finally, four known APs were identified without the use of certified standards (Table 2): AP-C in sample no. 11 (Figure S9), AP-F in samples no. 5 and 11 (Figure S10), ferintoic acid A in sample no. 12 (Figure S11), and oscillamide Y in samples no. 5 and 11 (Figure S12). For AP-F and oscillamide Y, two possible structures were found for each mass according to fragmentation spectra and the candidate list. However, due to the abundance of these two APs in toxic cyanobacterial blooms [7,19,44], they were identified as such, but were not confirmed with certified standards, so the identification is considered at level 2 [17,33].

### 2.5. Quantification and Semi-Quantification

The twelve samples from different locations in Canada, United Kingdom and France and underwent a quantitative analysis to monitor 17 known cyanotoxins (anatoxin-a (ANA-a), homoanatoxin-a (HANA-a), cylindrospermopsin (CYN), MCs: [Asp^3^]-LR, [Asp^3^]-RR, -LR, -RR, -YR, -LA, -LY, -LW, -LF, -WR, -HtyR and –HilR, AP-A and AP-B) according to previously published method [34]. Twelve cyanotoxins were reported in 11 lakes and results are shown in Table 3. For MCs, concentrations varied between 39 and 41,000 ng L^−1^ with MC-LR being the most abundant congener that was found in 67% of the samples. However, [Asp^3^]MC-RR and MC-RR were predominant in the two European samples (samples no. 11 and 12) with the highest concentrations being 41,000 and 5700 ng L^−1^ and MC-LA was also predominant in three samples (1, 2 and 4) with concentrations varying between 364 and 1165 ng L^−1^. In addition, AP-A and AP-B were found in half of the samples with concentrations varying from 95 up to 6000 ng L^−1^. These two APs were also predominant in two samples (5 and 6), and their ubiquity is supported by previous studies [5,34,45,46]. Finally, CYN was found in samples no. 8 at low concentration (153 ng L^−1^), but its mere presence is rather uncommon and can be linked to the evolution of cyanobacterial species and strains in relation with eutrophication and other stressors of ecosystems [47,48]. This high diversity of cyanotoxins present in these lakes is a marker of the potential diversity in strains of toxic cyanobacteria.

Indeed, 11 uncommon cyanotoxins were found in 42% of the samples (sample no. 2, 5, 9, 11 and 12) including four unreported MCs and APs. These compounds were semi-quantified in order to estimate their concentration levels in the samples (Table 4). Different reference materials were chosen for each semi-quantified compound, according to the similarities in terms of structure and physico-chemical proprieties. All concentrations varied between 57 and 1035 ng L^−1^ corresponding to MC levels lower, equal or higher to the proposed recommendations for MC-LR equivalents by the World Health Organization (WHO) (1 µg L^−1^) and by the U.S. EPA (0.3 to 1.6 µg L^−1^ for 10 days) for drinking water as a primary comparison for toxicity [4,49]. However, very little to no information is available about bioactivity and toxicity of these new compounds, making the assessment of risks quite difficult to evaluate public health and environmental impact, due to the presence of this diversity of cyanotoxins in lake water samples [5]. The study of compounds with lower toxicity is also relevant since the toxicology of the majority of these compounds is still not well understood, implying that the accumulating effects of bioactivity and the synergetic effects are also unknown. In the future, it would be interesting to deepen the understanding of cyanotoxins toxicity by studying samples contaminated by a variety of known and unknown cyanotoxins to understand the impact of a complex cyanobacterial bloom as a whole and to study the bioactivity of less known and unknown, but sometimes abundant, cyanotoxins individually.

## 3. Conclusions

In this study, a new suspect screening strategy, based on a DIA experiment, was developed for the unambiguous identification of uncommon microcystins and anabaenopeptins congeners. This DIA-based method was developed with an automated SPE coupled to UHPLC with heated electrospray ionization, and detection by a Q-Orbitrap^TM^, which allowed a sensitive and high-throughput analysis with minimum sample treatment for the target-screening of 17 cyanotoxins, and the suspect screening of MCs and APs in freshwater samples. A structural-based methodology supported by fragmentation spectra led to the characterization of 11 uncommon cyanotoxins, including two MCs ([Leu^1^, Ser^7^]MC-HtyR and [Asp^3^]MC-RHar) and two APs (AP731 and AP803), that have not yet been reported in the literature and were found in five of the twelve surface water samples from different lakes located in Canada, United Kingdom and France. These cyanotoxins were subsequently semi-quantified with levels of concentrations varying between 57 and 1035 ng L^−1^. Twelve targeted cyanotoxins were found in 11 lakes with concentrations ranging from 39 to 41,000 ng L^−1^. Overall, high diversity in terms of cyanotoxins and concentrations was observed, which highlights all the work still required on the discovery of cyanotoxins and the understanding of their impact on the environment. Finally, to our knowledge, this is the first report of a suspect screening strategy, based on a DIA experiment for the simultaneous identification and characterization of known and unknown MCs and APs. DIA experiment, has the advantage of providing more information in the fragmentation spectra than other common acquisition methods, but also makes it possible to quantify suspect compounds via the FS acquisition directly. Although suspect-screening methods can be time consuming for routine analysis when compounds are unknown, they can be very powerful for the identification of new structures. In addition, by targeting known and specific fragments, it would, therefore, be possible to use the developed method to characterize field cyanobacterial blooms by identifying uncommon cyanotoxins following a routine quantitative analysis when using available HRMS instruments. This method could eventually be applied to field samples and cultures to include other families of cyanopeptides to cover a larger range of cyanotoxins and ultimately perform a more accurate characterization of toxic algal blooms.

## 4. Materials and Methods 

### 4.1. Chemicals, Reagents and Stock Solutions 

ANA-a, CYN, MC-LR, [Asp^3^] MC-LR and MC-RR (purity ≥ 99%) were purchased from the National Research Council of Canada (Halifax, NS, Canada). Nodularin-R (NOD-R), [Asp^3^] MC-RR, MC-YR, LA, LY, LW, LF, WR, HtyR and HilR (purity ≥ 95%) were purchased from Enzo Life Science (Farmingdale, NY, USA). Homoanatoxin-a (HANA-a, purity ≥ 99%) was obtained from Abraxis, Inc. (Warminster, PA, USA), Anabaenopeptin A and B (AP-A, AP-B, purity ≥ 90%) from Cyano Biotech GmbH (Berlin, Germany), and ^15^N_10_-MC-LR (95%) from Cambridge Isotopes Laboratories, Inc. (Tewksbury, MA, USA). Individual stock solutions of ANA-a, CYN, MC-LR, [Asp^3^] MC-LR and MC-RR were kept at −20 °C for a maximum of six months. All other individual stock solutions were prepared in methanol (MeOH) at a concentration of 25 mg L^−1^ and were kept at −20 °C for a maximum of one year. Primary working solutions were prepared at a concentration of 100 µg L^−1^ for targeted cyanotoxins and 9 µg L^−1^ for internal standards (Iss: ^15^N_10_-MC-LR and NOD-R) by dilution in MeOH of individual stock solution aliquots. Subsequent working solutions were prepared daily by dilution in water to give solutions of the desired concentration. All organic solvents and water used for dilutions were of HPLC grade purity from Fisher Scientific (Whitby, ON, Canada). 

### 4.2. Sample Collection, Preparation and Quantification

Surface water sampling was conducted by the ATRAPP (Algal Blooms, Treatment, Risk Assessment, Prediction and Prevention through Genomics) research initiative co-financed by Genome Quebec and Genome Canada. The samples were collected in the photic zone of several lakes under surveillance, due to their occurrence of toxic algal blooms located in Canada, United Kingdom and France (Table S1). At each sampling location, a duplicate set of samples was collected in 125 mL amber polyethylene terephthalate glycol-modified (PETG) bottles (Thermo Scientific^TM^ Nalgene^TM^, Waltham, MA, USA), previously rinsed three times with the surface water from the site [34]. The bottles were then filled to the brim, sealed, stored at −20 °C until shipment and sent to the laboratory within 3 days. Upon reception at the laboratory, the samples underwent cell lysis to release the cyanotoxins with three freeze-thawing cycles. The samples were subsequently filtered through 25 mm diameter, 0.2 µm pore size Acrodisc GH Polypro (GHP) filters (Waters, Milford, MA, USA) [34]. A volume of 1450 µL of each filtered sample was transferred into 2-mL amber glass vials and kept at −20 °C until analysis. For all optimization experiments, analytes were spiked in water matrix consisting of analyte-free lake water sampled before harmful algal bloom seasons or matrix-matched water. Five replicates are spiked at mid-level concentration from linearity range (200 ng L^−1^). Prior to quantitative analysis, the internal standards were added for a final concentration of 300 ng L^−1^. Samples underwent a quantitative analysis to monitor 17 known cyanotoxins (ANA-a, HANA-a, CYN, MCs: [Asp^3^]-LR, [Asp^3^]-RR, -LR, -RR, -YR, -LA, -LY, -LW, -LF, -WR, -HtyR and -HilR, AP-A and AP-B) according to previously published method [34]. Samples with most interesting results (e.g., high cyanotoxins concentrations and the presence of less common congeners) were selected to conduct further suspect screening analysis. 

### 4.3. Instrumental Conditions

A Thermo Scientific Dionex UltiMate^TM^ 3000 RS pump and column compartment were used for chromatographic separation. The Dionex UltiMate^TM^ 3000 pump was coupled to the system used for on-line solid phase extraction (SPE), and both were controlled by Chromeleon 7.2 Software (Thermo Fisher Scientific, Waltham, MA, USA and Dionex Softron GMbH part of Thermo Fisher Scientific, Germering, Germany). A PAL system RTC autosampler was used (Zwingen, Switzerland) for injection. A Hypersil Gold (20 × 2mm, 12µm particle size, 175 Å pore size) column was used for on-line SPE, and the chromatographic separation was done with a Hypersil Gold (100 × 2.1mm, 1.9µm particle size, 175 Å pore size) column kept at 55 °C. Analysis of samples was performed using a Q-Exactive mass spectrometer controlled by the Xcalibur 3.0 software (Thermo Fisher Scientific, Waltham, MA, USA). Instrument calibration in positive mode was done every 7 days with a direct infusion of an LTQ Velos ESI Positive Ion Calibration Solution (Pierce Biotechnology Inc. Rockford, IL, USA), i.e., a mixture of caffeine, Met-Arg-Phe-Ala (MRFA) and Ultramark 1621 to reach mass accuracy within the 5 ppm range. Mass accuracy for all target compounds remained in the 5 ppm range in the 7-days post calibration.

#### 4.3.1. On-Line Solid Phase Extraction and Chromatographic Conditions

On-line SPE and chromatographic conditions were adapted from previous quantitative method [34]. Briefly, 1 mL of the sample was injected, and the loading speed from the injection loop to the SPE column was 1 mL min^-1^. A washing volume of 0.5 mL passed through the column following the sample loading step. The pre-concentration columns were finally back-flushed with MeOH and the eluting analytes were transferred using the analytical pump gradient directly through the analytical column and chromatographic separation is proceeded with the solvents acetonitrile (B), and water (A) with the addition of 0.1% formic acid at a flow rate of 525 µL min^-1^. A total chromatographic run of 8 min was carried out for the first screening step of the samples (quantitative analysis). The chromatographic run was extended to 30 min to ensure better chromatographic separation when using the suspect screening method via DIA mode. These chromatographic parameters were also applied to the samples, including calibration curve and quality control standards for semi-quantification (see Supporting Information Figure S13 for more details).

#### 4.3.2. HRMS Conditions

All the details about the HRMS conditions for quantitative analysis are presented in a previously published method [34]. The same ionization parameters were selected for the suspect screening acquisition method (see Table S5 for more details). For the DIA runs, each cycle consisted of one FS with resolving power set at 35,000 at full width at half maximum (FWHM) at *m/z* 200 with scan range between *m/z* 300 and 1400 to include singly and doubly charged ions from the MCs and APs suspect lists. The FS event was followed by 22 isolation scan windows acquired at a resolving power was set at 17,500 FWHM at *m/z* 200. Each isolation window width was set at *m/z* 50 and optimized to limit potential cofragmented ions, while getting enough acquisition points per chromatographic peaks [38]. NCE of 10, 20 and 30 were applied to ensure optimal fragmentation of suspect ions.

### 4.4. Suspect Screening Using DIA Methodology

The FS data were first processed using Compound Discoverer 3.0 (Thermo Fisher Scientific, Waltham, MA, USA). The workflow was built for the search of unknown compounds with in-house database searches, including all suspected cyanopeptides. MCs and APs were processed separately with the same workflow, but different database lists built according to the different molecular combinations, based on the potential amino acids in the molecules (Figure S1) [5,7,10,11,29]. The data processing consisted first of a spectra selection with a retention time filter between 4 min (lower limit) and 15 min (upper limit), a peak integration, a retention time alignment, an unknown compound detection, an isotope and adduct peak grouping (H^+^, Na^+^, K^+^), an unknown compound grouping and features merging, and a blank subtraction using uncontaminated lake water samples. Then, the grouped compounds were investigated in the in-house database searching with a mass tolerance of 5 ppm and retention time tolerance of 0.05 min. These databases were individually constructed for MCs and APs in Excel^®^ sheets, including the masses values from all the possible congener’s combinations minus the duplicates, which resulted in 8,709 individual masses for MCs and 8,815 individual masses for APs. Afterwards, a composition prediction was achieved, including minimum and maximum element counts (MCs: C_39_H_54_N_7_O_12_ to C_71_H_115_N_14_O_21_S_4_, APs: C_28_H_47_N_7_O_8_ to C_64_H_87_N_12_O_16_S_2_Br_2_Cl), and the maximum includes all possible elements present in all congeners. Finally, only the compounds detected in duplicate with a coefficient of variation of the signal intensity lower than 30% were retained for later steps. 

The following data treatment was performed using the Xcalibur 3.0 Software (Thermo Fisher Scientific, Waltham, MA, USA). MCs and APs have both few distinctive fragments, which were used to narrow down the list of features, and these were searched in the MS/MS spectra from the DIA acquisition. For MCs, the Adda function was the specific marker with two fragments simultaneously found in a MS/MS spectra: A first common to all congeners (*m/z* 163.11229-Adda-134-NH_3_+H^+^), and a second specific to the form of Adda (*m/z* 135.08099—Adda and (*6Z*)Adda, *m/z* 121.06534-DMAdda, *m/z* 163.07591-ADMAdda). For APs, the *m/z* 84.08136 fragment was used as a marker that corresponds to the immonium ion of lysine, an amino acid found in all APs. Though the mass is low, this fragment is rarely found in environmental samples when lysine is not present [50]. Finally, a second analysis of the samples was achieved in PRM scan mode with an inclusion list, including the exact masses from the last features list. Structural characterization was done with product ions and by associating this assignment with the amino acid combinations in the list of suspects generated for MCs and APs.

## Figures and Tables

**Table 1:** Detailed fragment and parent ions identified from MS/MS spectra and acquired using full scan (FS) and parallel reaction monitoring (PRM) scan modes of microcystins (MCs) candidates, with experimental fragment masses (*m/z*).

Parent and Fragment Ions	Known MC (Certified Standard)	Known MC (No Certified Standard)			Unknown MC	
	MC-LR	[GluOMe^6^] MC-LR	[M(O)^1^] MC-LR	[M(O)^1^, GluOMe^6^] MC-LR	[Asp^3^]MC-RHar	[Leu^1^, Ser^7^] MC-HtyR
M+H^+^	995.55927	1009.57104	1071.55340	1085.56928	1038.57291	1105.59150
Isotope #1	996.55629	1010.56808	1072.55682	1086.5709	1039.57413	1106.59113
Isotope #2	997.56067	1011.57490	1073.55194	1087.57513		1107.59307
Isotope #3	998.56488		1074.56341	1088.56762		1108.60241
M+2H^2+^			536.27992		519.78065	
Isotope #1			536.77931		520.29199	
Isotope #2			537.28075		520.79363	
Isotope #3					521.29156	
M+H^+^-H_2_O	977.56032	991.56022	1053.54316	1067.55874		
M+H^+^-CO	967.54996	981.57442				1077.59665
M+H^+^-CH_2_NHC(NH)NH_2_) (Arg)			999.49677			
M+H^+^-AA6		866.51198				976.54852
M+H^+^-134 (Adda)	861.47956	875.49571	937.48531	951.49270	904.49915	971.51668
M+H^+^-134 (Adda)-NH_3_	844.44971	858.46904	920.45409	934.46807	887.47064	954.49127
Z+Adda+AA6+AA3+AA1-CO+H^+^			847.43655	861.45323		
Z+Adda+AA6+AA3+H^+^	728.39793	742.41144	728.39593	742.41195	728.39601	728.39614
AA3+Z+Adda+AA6-H_2_O+H^+^	710.38705	724.40291	710.38457	724.40284		710.38447
Z+Adda+AA6+CO+H^+^	625.33379	639.34874	625.33299	639.34839		625.33274
AA3+Z+Adda+H^+^	599.35556	599.35522	599.35471	599.35514	599.35420	599.36213
Z+Adda+AA6+H^+^	599.35556	613.36953	599.35471	613.36946	613.37004	599.36213
AA3+Z+Adda-CO+H^+^	571.35843	571.35829	571.35844	571.35823	571.35963	571.36199
Z+Adda+AA6-CO+H^+^	571.35843	585.37421	571.35844	585.37418	585.37388	571.36199
[AA7+AA1+X+AA3+Z+NH_2_+2H]^+^	570.33513	570.33402	646.33282	646.33296	613.35189	680.37043
AA7+AA1+X+AA3+Z+H^+^	553.31097	553.30853	629.30526	629.30531	596.32510	663.34577
AA7+AA1+X+AA3+Z-H_2_O+H^+^	535.29685	535.29715	611.29594	611.29603	578.31403	
AA7+AA1+X+AA3+Z-CO+H^+^	525.31401	525.31395	601.31109	601.31177	568.33044	635.35001
[AA1+X+AA3+Z+NH_2_+2H]^+^		487.29752	563.29411	416.26012		593.34189
AA1+X+AA3+Z+H^+^	470.26987	470.26974	546.26878	546.27001	513.28930	576.31199
AA1+X+AA3+Z-NH_3_+H^+^	453.23973	453.23985	529.24225	529.24229	496.26170	559.28760
AA1+X+AA3+Z-H_2_O+H^+^	452.25983	452.25967	528.25943	528.25977	495.26546	
AA1+X+AA3+Z-CO-NH_3_+H^+^					468.26542	
Z+Adda-134+AA6-NH_3_+H^+^	448.25002	462.27024	448.25379	462.27020	462.27011	
Adda-134+AA6+AA7+AA1-NH_3_+H^+^	446.22694	460.24297	522.22418	536.24198		492.27009
[X+AA3+Z+NH_2_+2H]^+^		416.26101		416.26113		
X+AA3+Z+H^+^	399.23512	399.23409	399.23411	399.23417	442.25185	463.22949
AA7+AA1+X+AA3+H^+^	397.20653	397.20649			426.20885	507.24592
AA6+AA7+AA1+X+H^+^	397.20653	411.22257	473.20550	487.22124	440.22463	507.24592
X+AA3+Z-NH_3_+H^+^	382.20854	382.20868	382.20836	382.20855	425.22561	446.20174
Adda-134+AA6+AA7-NH_3_+H^+^	375.19117	389.20689	375.19028	389.20690	375.19269	379.18595
Adda-134+AA6+AA7-NH_3_-CO+H^+^	347.19498	361.21108		361.21113	347.19155	351.19024
Adda-134+AA6-NH_3_+H^+^	292.15384	306.16894	292.15371	306.16887		292.15414
[AA3+Z+NH_2_+2H]^+^		303.17697	303.17668	303.17683	303.17739	303.17752
AA7+AA1+X-NH_3_+H^+^					294.15521	
X+AA3+H^+^					272.13442	307.12806
AA3+Z-NH_2_+H^+^	286.14888	286.14981	286.14997	286.14989	286.14832	286.14935
AA7+AA1+X+H^+^	268.16531	268.16581	344.16287	344.16366	311.18244	378.20111
AA1+Z+H^+^					242.16093	
AA6+AA7+CO^+^		253.08124		253.08129	239.06653	243.06043
AA6+AA7+H^+^	213.08659	227.10269	213.08735	227.10254	213.08693	217.08173
AA6+AA7-CO+H^+^					185.09586	189.08683
[Z+NH_2_+2H]^+^		174.13423		174.13431		174.13459
Adda-134-NH_3_+H^+^	163.11149	163.11151	163.11156	163.11148	163.11138	163.11150
AA7+AA1+H^+^	155.08136	155.08127	231.07989	231.07983	155.08138	201.12293
AA7+AA1-CO+H^+^	127.08639	127.08636	203.08461	203.08484	127.08664	173.12822
Adda frag (Ph-CH_2_-CH(O^+^Me)	135.08040	135.08041	135.08073	135.08055	135.08049	135.08049
X Immonium ion		86.09695	86.09680	86.09682	129.11388	129.11358
Ser Immonium ion						60.04481
Leu Immonium ion	86.09677					86.09689

**Table 2:** Detailed fragment and parent ions identified from MS/MS spectra and acquired using FS and PRM scan modes of each anabaenopeptin (AP) candidates, with experimental fragment masses (*m/z*).

Parent and Fragment Ions	Known AP (Certified Standard)	Known AP (No Certified Standard)				Unknown AP	
	AP-A	AP-C	AP-F	Ferintoic acid A	Oscillamide Y	AP731	AP803
M+H^+^	844.42399	809.45396	851.47649	867.43760	858.43789	732.39224	804.43535
Isotope #1	845.42487	810.45755	852.47948	868.44117	859.44270	733.39499	805.43704
Isotope #2	846.42939	811.46099	853.48112	869.44461	860.44575	734.39684	806.43070
Isotope #3	837.43126				861.44882	735.39821	807.43308
M+H^+^-NH_3_		792.42755					
M+H^+^-H_2_O	826.41253	791.44322	833.46495	849.42755	840.42805	714.38127	786.42485
M+H^+^-H_2_O-CO				821.43177			758.42793
M+H^+^-AA6_residue_						603.34925	657.39861
M+H^+^-AA1				681.36103	695.37553		
M+H^+^-AA1-H_2_O	663.34863	663.34859	677.36401	663.34841	677.36411	567.31335	673.33952
M+H^+^-CO-AA1-H_2_O	635.35366	635.35363		635.35349	649.37013	539.31810	645.34572
M+H^+^-AA4-AA5	528.28961	547.32259	589.34485	605.30624	596.30578	548.26999	564.25123
M+H^+^-AA3-AA4				591.29012			
M+H^+^-AA3-AA4-H_2_O	550.26467	515.29601		573.28032	550.26427	502.22842	542.29930
M+H^+^-AA3-AA4-CO	540.28143			563.29597	540.28113		532.31511
M+H^+^-AA1-CO-AA6_residue_-H_2_O						479.29701	581.34602
M+H^+^-AA1-CO-AA6_resisue_						428.28596	516.32042
M+H^+^-AA1-AA4-AA6_residue_-H_2_O						394.20841	496.25720
M+H^+^-AA1-AA3-AA4	405.21182	405.21189	405.21165	405.21192	405.21185	373.17124	447.22582
Lys+AA3+AA5+AA6+H^+^	460.29013		474.30651	460.28997	474.30632	428.24910	534.27652
Lys+AA5+AA6+CO+H^+^	389.21756			389.21743		357.17623	431.23184
AA6+Lys+CO+AA3+H^+^	403.23383			403.23379		385.20775	435.17195
AA3+AA4+AA5+H^+^	362.20673	362.20676	376.22245	362.20651	376.22195	284.19651	372.23107
AA5+AA6+H^+^	233.12808	233.12801	233.12811	233.12811	233.12809	201.08693	275.14190
AA3+AA4+H^+^	277.15417			277.15409	277.15416	213.16084	245.13166
AA4+AA5+H^+^	263.13861	263.13851	263.13865	263.13866	263.13866	185.12842	241.19079
AA1+H^+^			175.11875				
AA1+CO^+^			201.09792				
[AA1+2H]^+^		130.11017					
Ph-CH_2_-OH	107.04936				107.04913	107.04945	107.04961
Lys Immonium ion	84.08123	84.08122	84.08120	84.08119	84.08120	84.08134	84.08131
AA1 Immonium Ion	136.07545		129.11359			120.08070	86.09692
Phe Immonium		120.08100	120.08110		120.08110		

**Table 3:** Cyanotoxins detection in lakes from Canada, United Kingdom and France. Concentrations are reported in ng L^−1^ with a standard deviation of duplicate analysis (ND: Analyte not detected). * Indicative values ± concentration between method detection limit (MDL) and method quantification limit (MQL), which were previously reported by Roy-Lachapelle et al. (2019) [34]. Only the analytes with results > MDL are presented.

Sample No.	CYN	[Asp^3^]MC-RR	MC-RR	MC-YR	MC-LR	[Asp^3^]MC-LR	MC-HiIR	MC-LA	MC-LY	AP-A	AP-B
1	ND	ND	ND	ND	90 ± 28	ND	ND	486 ± 105	ND	ND	ND
2	ND	ND	ND	ND	ND	ND	ND	364 ± 70	ND	ND	95 ± 17
3	ND	ND	491 ± 95	76 ± 6	1010 ± 21	ND	ND	ND	ND	ND	ND
4	ND	ND	ND	ND	106 ± 10	ND	ND	1165 ± 60	ND	ND	ND
5	ND	ND	ND	ND	47 ± 5*	ND	ND	ND	ND	1290 ± 259	851 ± 116
6	ND	ND	ND	ND	ND	ND	ND	ND	ND	188 ± 66	348 ± 38
7	ND	ND	ND	ND	254 ± 29	ND	ND	ND	41 ± 13 *	ND	124 ± 32
8	153 ± 66	ND	ND	ND	62 ± 5	ND	ND	ND	ND	ND	ND
9	ND	ND	ND	ND	ND	ND	ND	ND	ND	ND	ND
10	ND	ND	ND	ND	ND	ND	ND	ND	ND	ND	ND
11	ND	41,364 ± 3885	840 ± 87	259 ± 17	416 ± 39	1073 ± 116	ND	ND	ND	3178 ± 97	5836 ± 187
12	ND	123 ± 8	5691 ± 506	2692 ± 382	3263 ± 179	ND	321 ± 98	ND	39 ± 36 *	137 ± 25	239 ± 46

**Table 4:** MCs and APs identified in samples with semi-quantified concentration levels reported in ng L^−1^ with a standard deviation of duplicate analysis (ND: Analyte not detected).

Sample No.	[GluOMe^6^] MC-LR	[M(O)^1^] MC-LR	[M(O)^1^, GluOMe^6^] MC-LR	[Asp^3^] MC-RHar	[Leu^1^, Ser^7^] MC-HtyR	AP-C	AP-F	Ferintoic acid A	Oscillamide Y	AP731	AP803
3	596 ± 36	57 ± 11	197 ± 28	ND	ND	ND	ND	ND	ND	ND	ND
5	ND	ND	ND	ND	ND	ND	175 ± 34	ND	484 ± 55	ND	1035 ± 108
9	ND	ND	ND	201 ± 47	ND	ND	ND	ND	ND	ND	ND
11	ND	ND	ND	ND	ND	75 ± 9	221 ± 15	ND	88 ± 10	109 ± 7	ND
12	ND	ND	ND	ND	124 ± 23	ND	ND	60 ± 11	ND	ND	ND

**NB.** Reference material for semi-quantification were chosen as the follow: MC-LR for [GluOMe^6^]MC-LR, [M(O)^1^]MC-LR and [M(O)^1^, GluOMe^6^]MC-LR; [Asp^3^]MC-RR for [Asp^3^]MC-RHar; MC-HtyR for [Leu^1^, Ser^7^]MC-HtyR; AP-B for AP-C and AP-F; AP-A for ferintoic acid A, oscillamide Y, AP731 and AP803.

## Supplementary Materials

The following are available online at https://www.mdpi.com/2072-6651/11/11/619/s1, Figure S1: Configuration of amino acids (AA) in MCs and APs, Table S1: Details on samples with sampling date and region of sampling in Canada and Europe, Table S2: Confidence of identification by levels and number of features obtained at each step of identification using Compound Discoverer 3.0 software, Figure S2: Chromatogram, isotopic pattern and fragmentation spectra of MC-LR with RT at 8.79 min, Table S3: Number of MCs combinations using fragmentation spectra and identification of amino acids, Figure S3: Chromatogram, isotopic pattern and fragmentation spectra of feature *m/z* 1009.57104 identified as [GluOMe^6^]MC-LR with RT at 11.95 min, Figure S4: Chromatogram, isotopic pattern and fragmentation spectra of feature *m/z* 1071.55340 identified as [M(O)^1^]MC-LR with RT at 7.50 min, Figure S5: Chromatogram, isotopic pattern and fragmentation spectra of feature *m/z* 1085.56928 identified as [M(O)^1^, GluOMe^6^]MC-LR with RT at 8.11 min, Figure S6: Chromatogram, isotopic pattern and fragmentation spectra of feature *m/z* 1038.57291 identified as [Asp^3^]MC-RHar with RT at 7.37 min, Figure S7: Chromatogram, isotopic pattern and fragmentation spectra of AP-A with RT at 7.40 min, Table S4: Number of AP combinations at each level of identification using fragmentation spectra and identification of amino acid, Figure S8: Chromatogram, isotopic pattern and fragmentation spectra of feature *m/z* 732.39224 identified as AP731 with RT at 6.53 min, Figure S9: Chromatogram, isotopic pattern and fragmentation spectra of feature *m/z* 809.45396 identified as AP-C with RT at 7.31 min, Figure S10: Chromatogram, isotopic pattern and fragmentation spectra of feature *m/z* 851.47649 identified as AP-F with RT at 7.05 min, Figure S11: Chromatogram, isotopic pattern and fragmentation spectra of feature *m/z* 867.4376 identified as ferintoic acid A with RT at 7.72 min, Figure S12: Chromatogram, isotopic pattern and fragmentation spectra of feature *m/z* 858.43789 identified as oscillamide Y with RT at 7.58 min, Figure S13: Details on the on-line SPE – UHPLC chromatographic gradient program for quantification (a) and suspect screening methods (b), Table S5: Ionization and HRMS acquisition parameters.
