# Supplementary material for: A Data-Independent Methodology for the Structural Characterization of Microcystins and Anabaenopeptins Leading to the Identification of Four New Congeners
Source: Toxins (Basel). 2019 Oct 26;11(11):619. doi: 10.3390/toxins11110619 (PMC6891544; doi:10.3390/toxins11110619)
Supplement: Supplementary file 1 [file toxins-11-00619-s001.pdf]

Supplementary Materials: A Data-Independent Methodology for the Structural Characterization of Microcystins and Anabaenopeptins Leading to the Identification of Four New Congeners

Audrey Roy-Lachapelle, Morgan Solliec, Sébastien Sauvé and Christian Gagnon

Microcystins						
**AA1**	**X**	**AA3**	**Z**	**AA5**	**AA6**	**AA7**
Ala	Leu	MeAsp	Arg	Adda	Glu	Mdha
Leu	Arg	Asp	Glu(OMe)	ADMAdda	Glu(OMe)	Dha
Ser	Tyr		Ala	6(Z)-Adda	Glu(OC3H7)	Dhb
Gly	Hty		Tyr	DMAdda		L-Ser
MeAla	Glu(OMe)		Har			L-MeSer
MeLeu	Phe		Phe			(Z)-Dhb
Met	Glu		Trp			L-MeLan
Met(O)	Hil		Leu			Mdhb
Phe	Trp		Aba			Thr
	(H4)Tyr		Met			
	Ala		Hty			
	Hph		Met(O)			
	Met(O)		Val			
	Val		Bu			
	Aha		Apa			
	Met(O2)		Hph			
	Har		Tyr(OMe)			
	MeHty					
	Hty(OMe)					
	Tyr(OMe)					

Abaenopeptins						
**AA1**	**CO**	**Lys**	**AA3**	**AA4**	**MeAA5**	**AA6**
Tyr			Val	Hty	MeAla	Phe
Arg			Ala	Hph	MeIle	Ile
Ile			Ile	Hty(OMe)	MeHty	Leu
Leu			MeIle	Leu	Cl,MeTrp	Ser
Phe			Leu	MeHph	MeGly	AcSer
Lys			Met	EtHph	MeHph	BrTrp
Trp			Met(O)	MeHty	MeLeu	Tyr
			Met(O2)		MeAsn	Met(O)
					Br,MeTrp	Met
					MeCht	hTrp
					MehTrp	Hty
					MeAhpha	BhTrp
					MeCTrp	

Diminutive	Name
6(Z)-Adda	6(Z)-3-amino-9-methoxy-2,6,8-trimethyl-10-phenyl-4,6-decadienoic acid
(H4)Tyr	1,2,3,4-tetrahydrotyrosine
(Z)-Dhb	Dehydrobutyric acid
Aba	Amino-isobutyric acid
AcSer	Acetylserine
Adda	3-amino-9-methoxy-2,6,8-trimethyl-10-phenyl-4,6-decadienoic acid (2S,3S,8S,9S)-
ADMAdda	3-amino-9-acetoxy-2,6,8-trimethyl-10-phenyldeca-4,6-dienoic acid
Aha	Aminoheptaonic acid
Ala	Alanine
Apa	Aminopenicillanic acid
Arg	Arginine
Asp	Aspartic acid
BhTrp	Bromo hydroxy tryptophan
Br,MeTrp	Bromo methyl tryptophan
BrTrp	Bromo triptophan
Bu	Butyric Acid
Cl,MeTrp	Chloro methyl tryptophan
Dha	Dehydroalanine
Dhb	Dehydrobutyric acid
DMAdda	Desmethyl-Adda
EtHph	Ethyl homophenylalanine
Glu	Glutamic acid
Glu(OC3H7)	Glutamate ethyl ester
Glu(OMe)	Glutamate methyl ester
Gly	Glycine
Har	Homoarginine
Hil	Homoisoleucine
Hph	Homophenylalanine
hTrp	Hydroxy tryptophan
Hty	Homotyrosine
Hty(OMe)	Homotyrosine methyl ester
Ile	Isoleucine
Leu	Leucine
L-MeLan	*N* -methyl-lanthionin
Lys	Lysine
Mdha	N-methyl-dehydroalanine
Mdhb	Methyl-dehydrobutyric acid
MeAhpha	*N* -methyl-2-amino-6-(4′-hydroxyphenyl)hexanoic acid
MeAla	Methyl alanine
MeAsn	Methyl asparagine
MeAsp	Methyl aspartic acid
MeCht	Chloro hydroxy methyltryptophan
MeGly	Methyl glycine
MeHph	Methyl homophenylalanine
MehTrp	Hydroxy methyltryptophan
MeHty	Methyl homotyrosine
MeIle	Methyl isoleucine
MeLeu	Methyl leucine
MeSer	Methyl serine
Met	Methionine
Met(O)	Methionine sulfoxide
Met(O2)	Methionine sulfoxides
Phe	Phenylalanine
Ser	Serine
Trp	Tryptophan
Thr	Threonine
Tyr	Tyrosine
Tyr(OMe)	Methyl tyrosinate
Val	Valine

Figure S1. Configuration of amino acids (AA) in MCs and APs.

Table S1. Details on samples with sampling date and region of sampling in Canada and Europe.

Sample No.	Sampling Date	Region of Sampling
1	07-2017	Durham, ON
2	08-2017	Laurentides, QC
3	08-2017	York, ON
4	08-2017	Simcoe County, ON
5	08-2017	Wentworth County, ON
6	08-2017	Chaudière-Appalaches, QC
7	08-2017	Chaudière-Appalaches, QC
8	08-2017	Montérégie, QC
9	08-2017	Digby County, NS
10	09-2017	Chaudière-Appalaches, QC
11	08-2017	United Kingdom
12	09-2017	France

Table S2. Confidence of identification by levels and number of features obtained at each step of identification using Compound Discoverer 3.0 software.

Sample No.	Features	Exact Mass (Level 5)		Isotopes, Adducts, RT [1], Molecular Formula, SD [2] (Level 3)		Distinctive Fragments (Level 3)		Confirmed with Fragmentation (Level 2)	
		MCs	APs	MCs	APs	MCs	APs	MCs	APs
1	4,286	23	53	14	20	4	0	2	0
2	4682	107	83	47	41	12	1	2	0
3	5983	65	101	37	51	17	5	6	0
4	4212	45	78	21	28	3	6	2	0
5	4370	54	95	28	32	6	8	1	5
6	3173	47	60	3	24	3	3	0	2
7	3245	52	65	29	19	9	1	1	1
8	4027	116	92	34	26	7	2	2	0
9	4171	51	74	23	21	11	1	1	0
10	2960	38	49	18	21	10	2	1	1
11	1576	12	42	9	19	5	8	5	6
12	5909	102	104	18	38	10	7	7	3

[1] Retention time [2] Standard deviation.

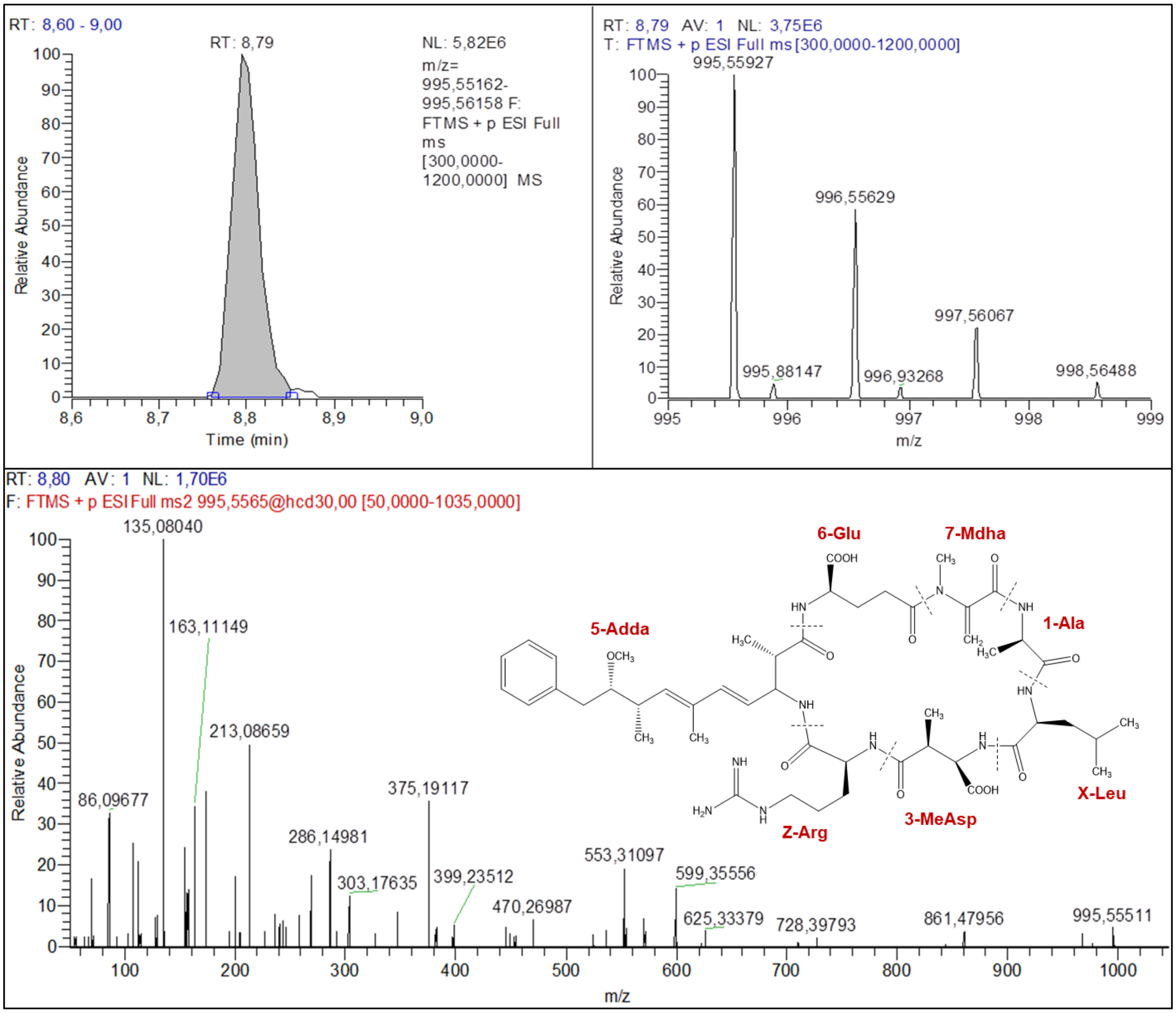

Figure S2. Chromatogram, isotopic pattern and fragmentation spectra of MC-LR with RT at 8.79 min.

Table S3. Number of MCs combinations using fragmentation spectra and identification of amino acids.

Identification [1]	[GluOMe[6]]MC-LR		[M(O)[1]]MC-LR		[M(O)[1], GluOMe[6]] MC-LR		[Asp[3]]MC-RHar		[Leu[1], Ser[7]]MC-HtyR	
	**Comb.** [2]	**Amino Acids** [3]	**Comb.**	**Amino Acids**	**Comb.**	**Amino Acids**	**Comb.**	**Amino Acids**	**Comb.**	**Amino Acids**
Exact mass	1316		856		823		934		482	
ADDA	878	ADDA<br>(6Z)Adda	434	ADDA<br>(6Z)Adda	408	ADDA<br>(6Z)Adda	636	ADDA<br>(6Z)Adda	300	ADDA<br>(6Z)Adda
Amino acid 6	322	GluOMe	142	Glu	142	GluOMe	170	Glu	160	Glu
Z	82	Arg	42	Arg	42	Arg	28	Har	48	Arg
Amino acid 3	42	MeAsp	20	MeAsp	20	MeAsp	18	Asp	24	MeAsp
X	10	Leu	6	Leu	6	Leu	10	Arg	4	Hty
Amino acid 1	6	Ala	6	M(O)	6	M(O)	6	Ala	2	Leu
Amino acid 7	6	Mdha<br>Dhb<br>(Z)Dhb	6	Mdha<br>Dhb<br>(Z)Dhb	6	Mdha<br>Dhb<br>(Z)Dhb	6	Mdha<br>Dhb<br>(Z)Dhb	2	Ser

[1] Identification level: exact mass and different amino acids identified using the MS/MS spectra.[2] Number of possible MCs combinations after each level of identification. [3] Identified amino acids at each site of the MC structure.

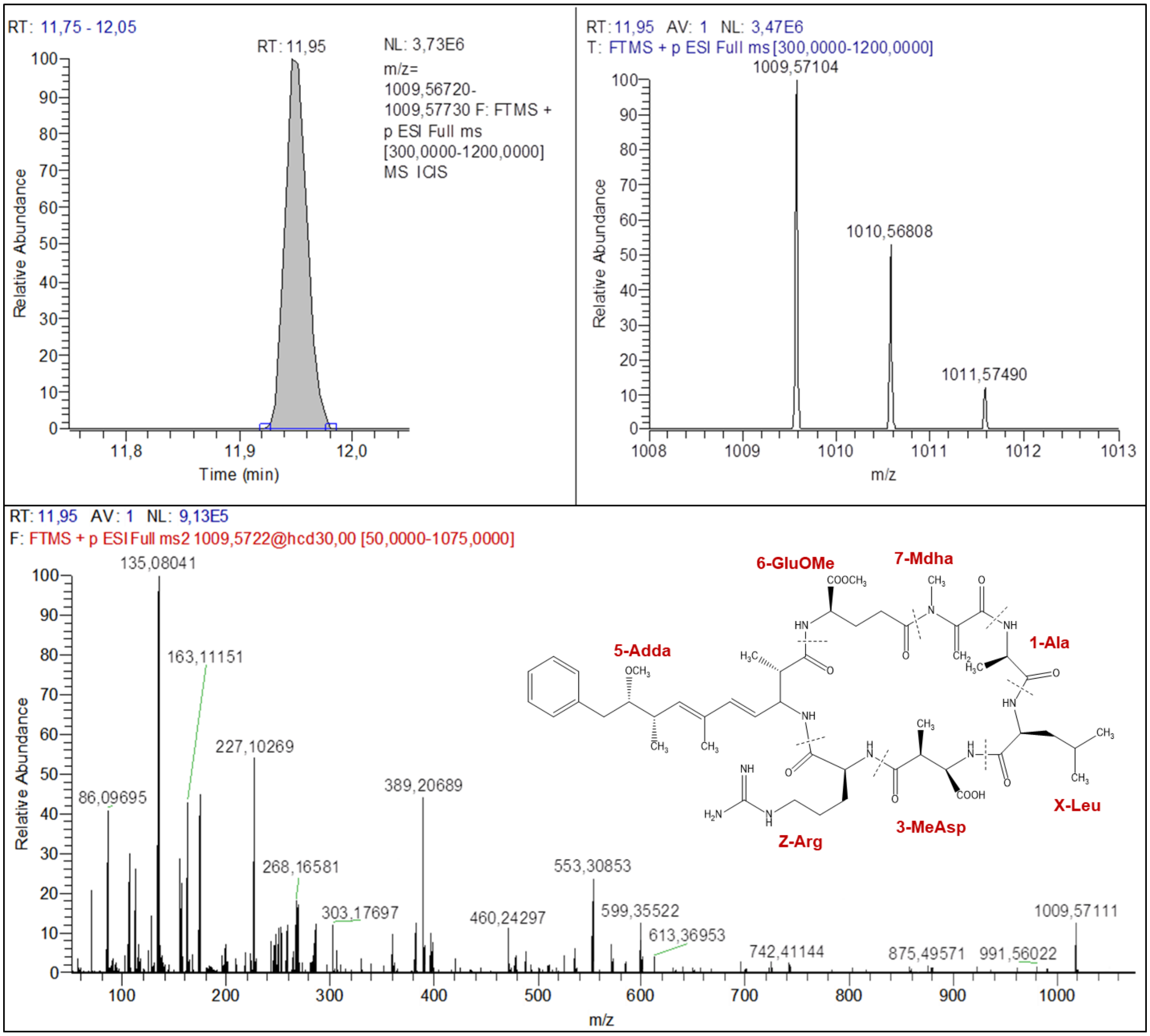

Figure S3. Chromatogram, isotopic pattern and fragmentation spectra of feature *m*/*z* 1009.57104 identified as [GluOMe[6]]MC-LR with RT at 11.95 min.

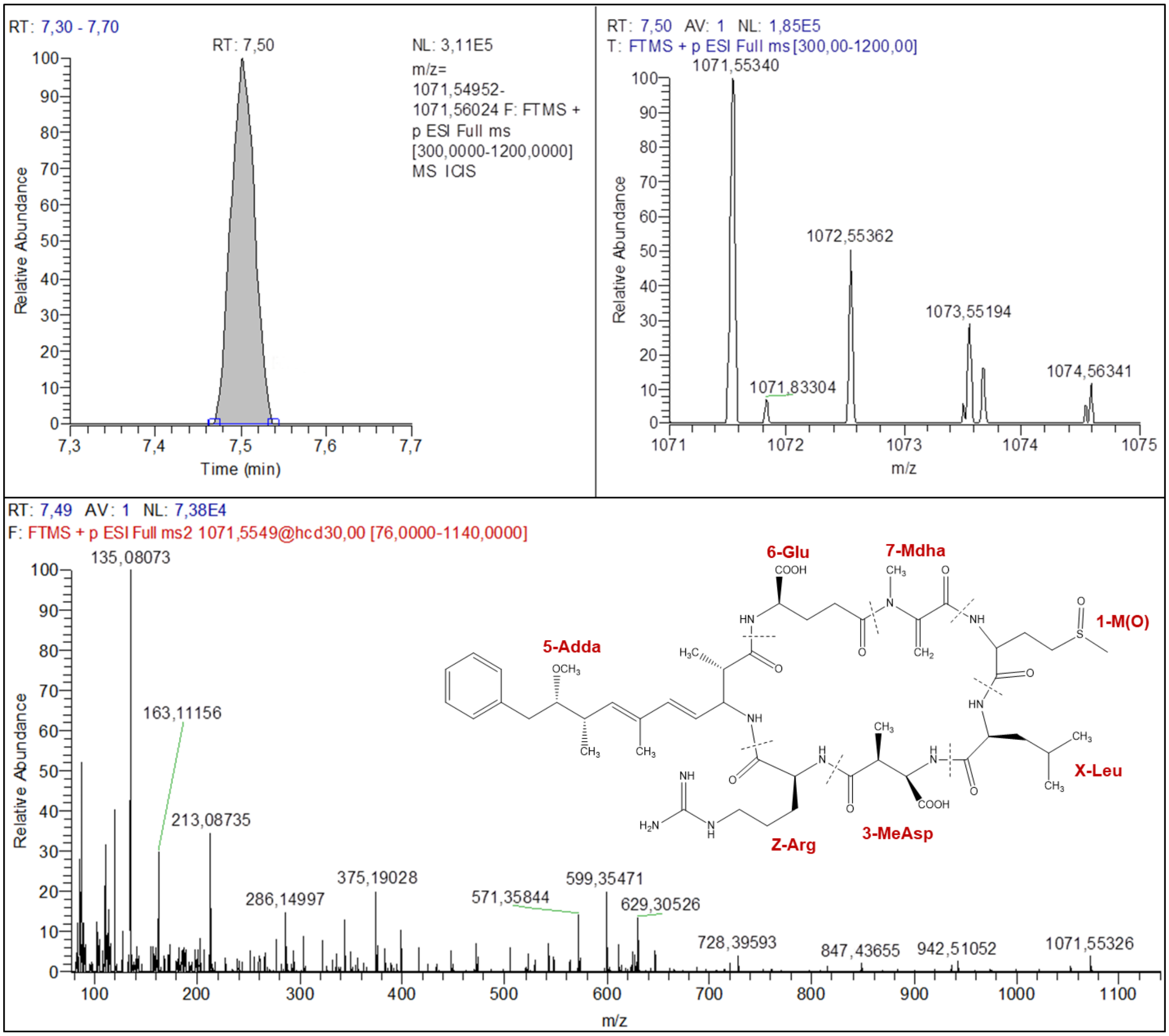

Figure S4. Chromatogram, isotopic pattern and fragmentation spectra of feature *m/z* 1071.55340 identified as [M(O)[1]]MC-LR with RT at 7.50 min.

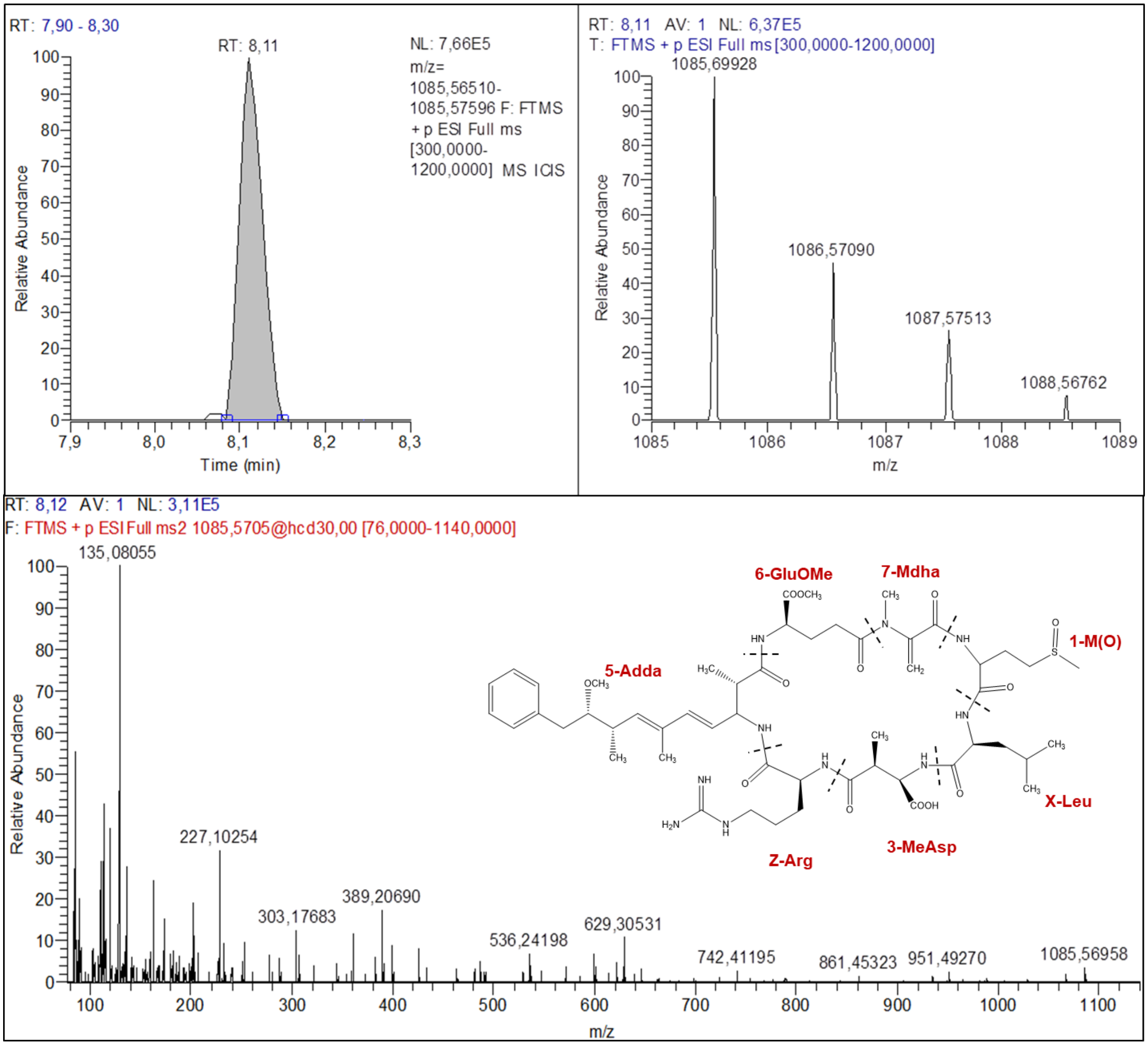

Figure S5. Chromatogram, isotopic pattern and fragmentation spectra of feature *m/z* 1085.56928 identified as [M(O)[1], GluOMe[6]]MC-LR with RT at 8.11 min.

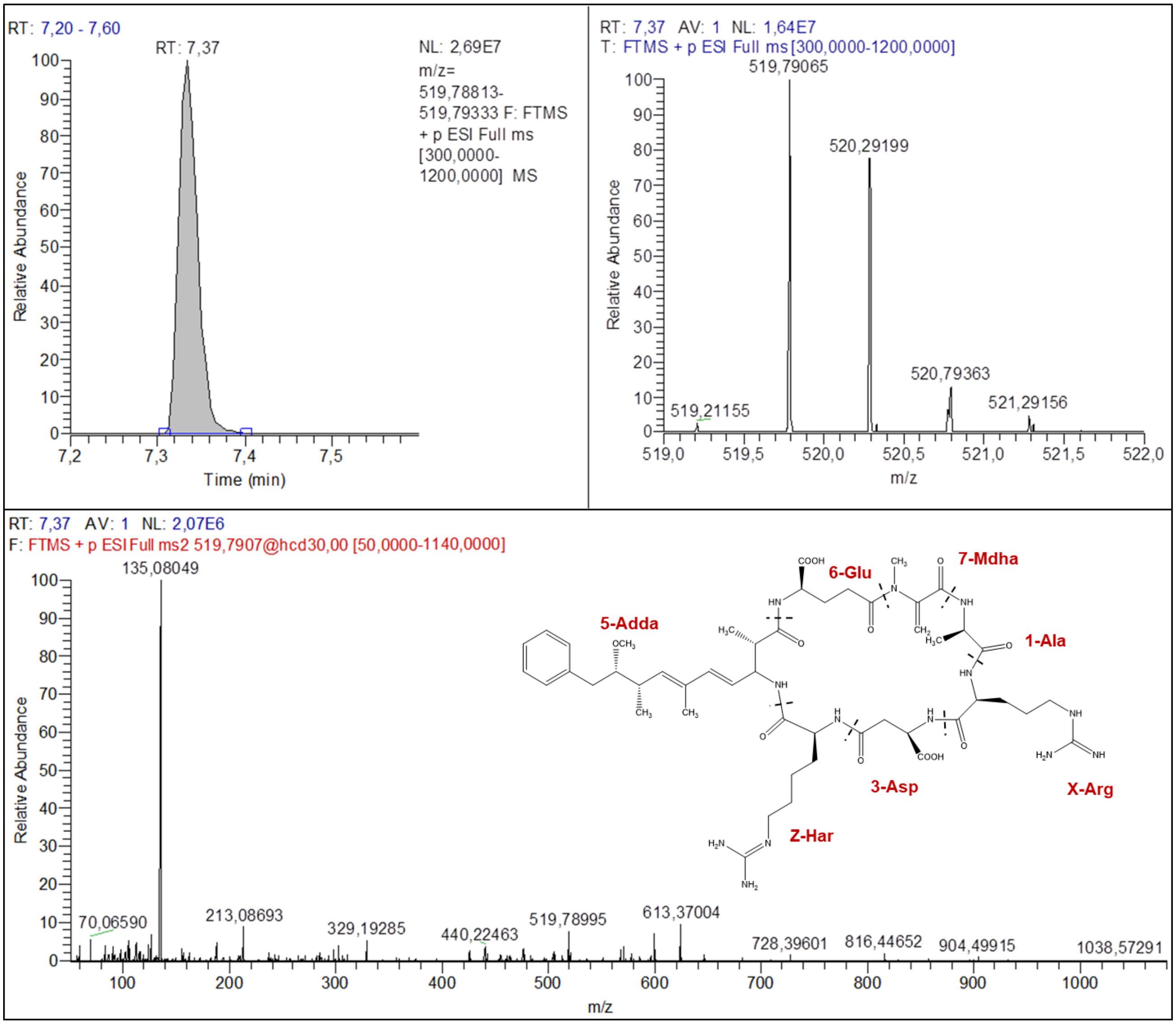

Figure S6. Chromatogram, isotopic pattern and fragmentation spectra of feature *m/z* 1038.57291 identified as [Asp^3]MC-RHar with RT at 7.37 min.

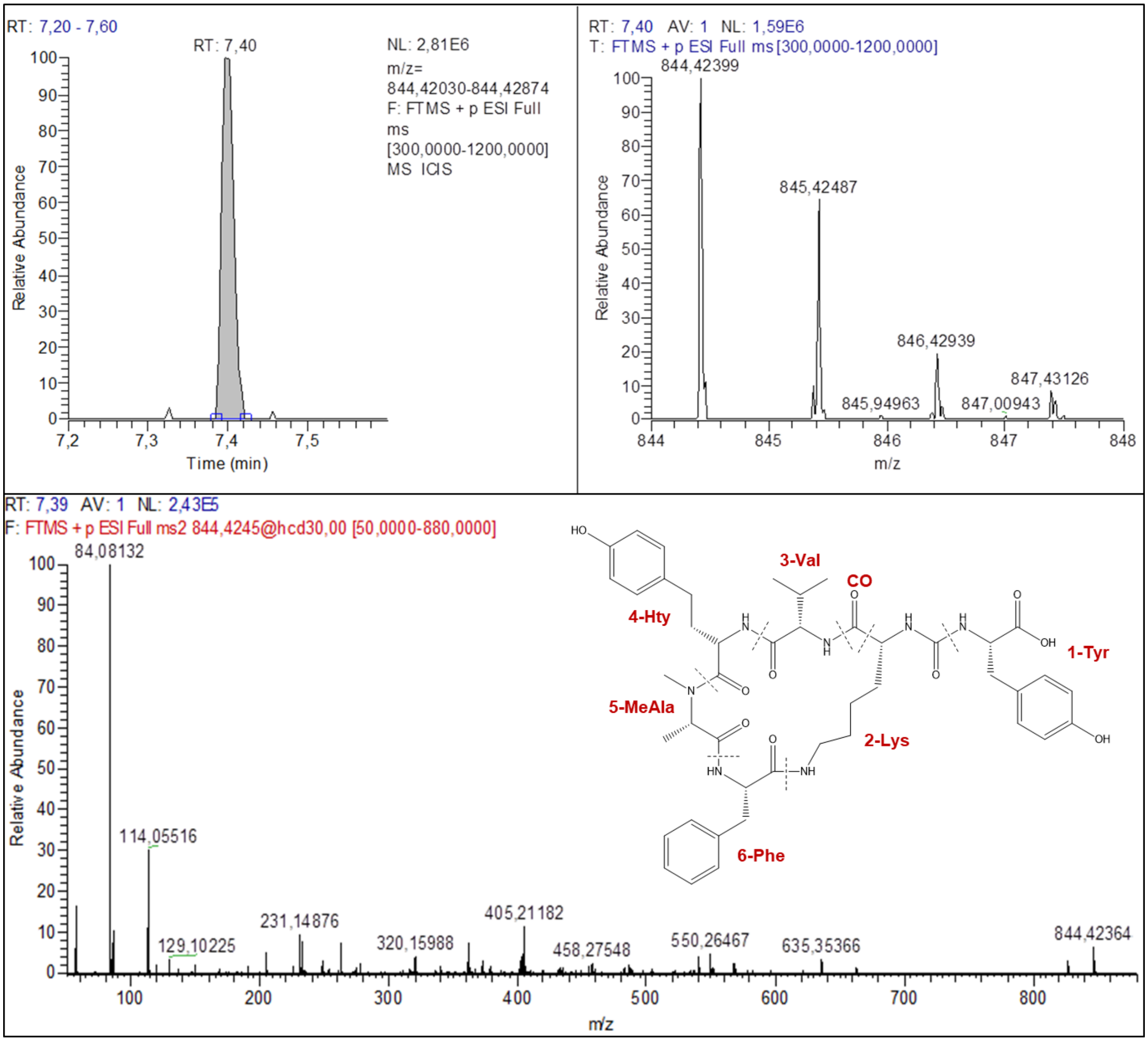

Figure S7. Chromatogram, isotopic pattern and fragmentation spectra of AP-A with RT at 7.40 min.

Table S4. Number of AP combinations at each level of identification using fragmentation spectra and identification of amino acids.

Identification [1]	AP-C		AP-F		Ferintoic acid A		Oscillamide Y		AP731		AP803	
	Comb. [2]	Amino Acids [3]	Comb.	Amino Acids	Comb.	Amino Acids	Comb.	Amino Acids	Comb.	Amino Acids	Comb.	Amino Acids
Exact mass	13		56		31		43		5		11	
Amino acid 1	13	Leu Ile	26	Arg	13	Trp	26	Tyr	1	Phe	8	Leu Ile
Amino acid 3	2	Val	2	Leu Ile	1	Val	2	Leu Ile	1	Val	2	Met

[1] Identification level: exact mass and different amino acids identified using the MS/MS spectra.[2] Number of possible APs combinations after each level of identification. [3] Identified amino acids at each site of the AP structure.

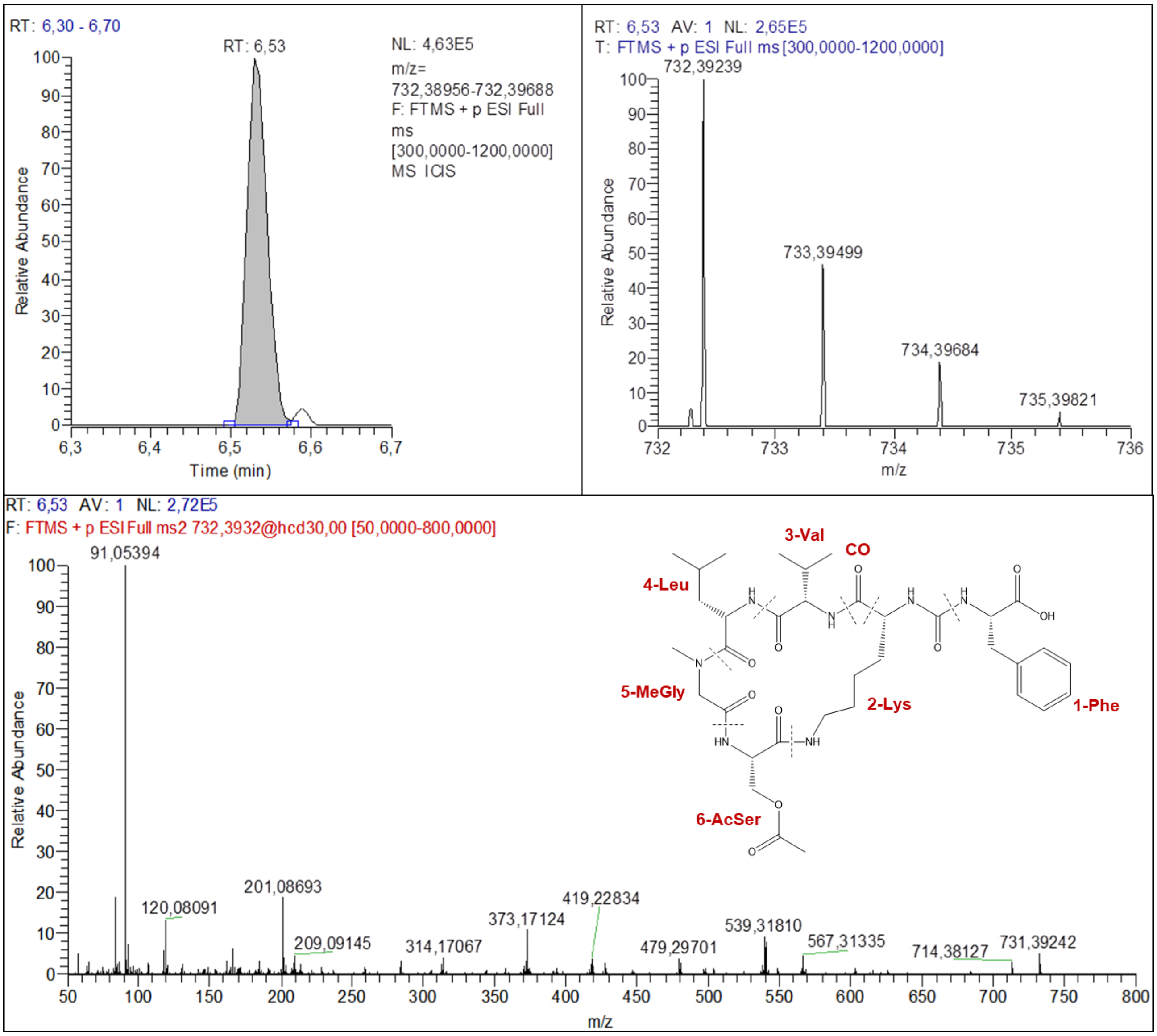

Figure S8. Chromatogram, isotopic pattern and fragmentation spectra of feature *m/z* 732.39224 identified as AP731 with RT at 6.53 min.

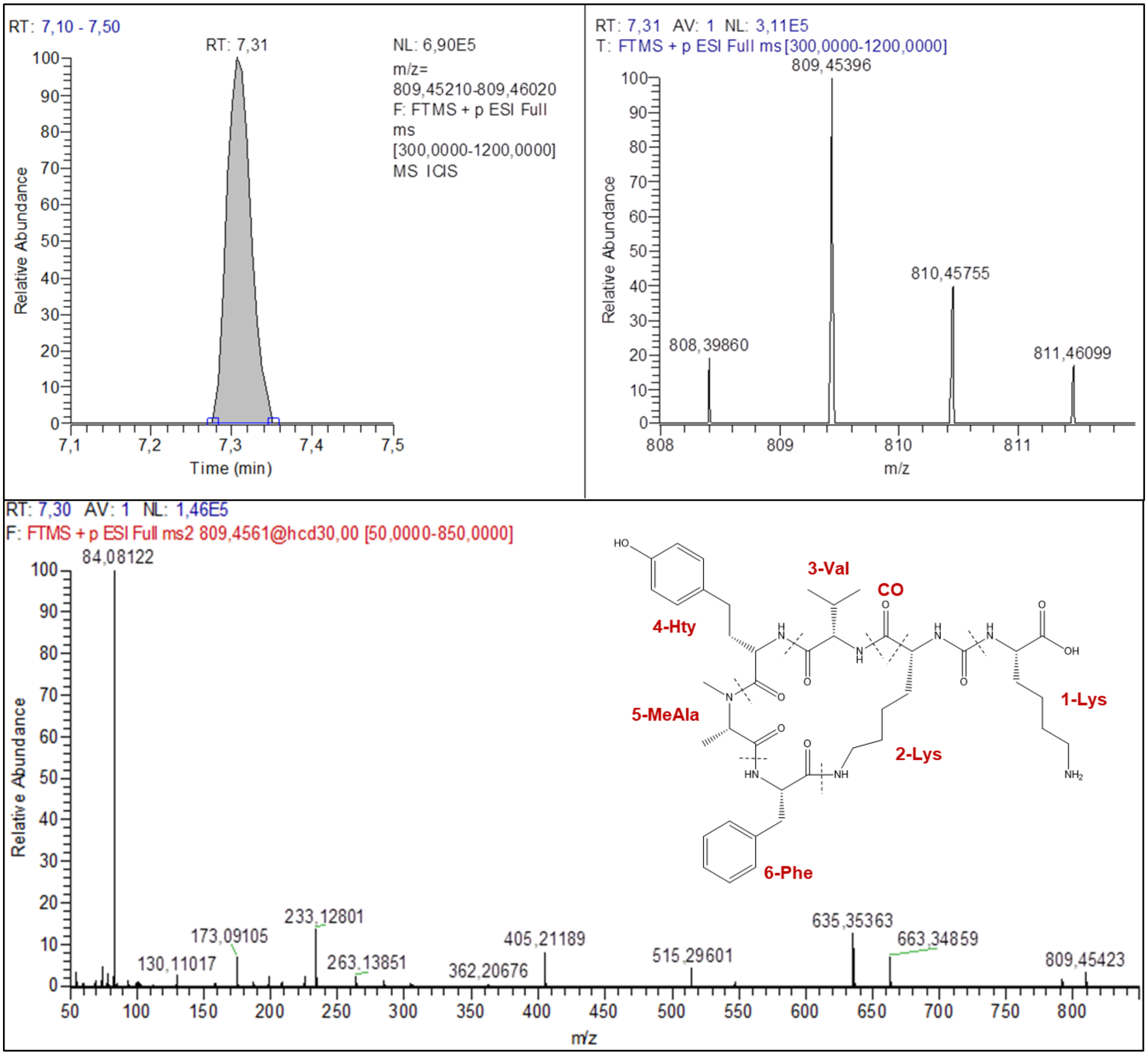

Figure S9. Chromatogram, isotopic pattern and fragmentation spectra of feature *m/z* 809.45396 identified as AP-C with RT at 7.31 min.

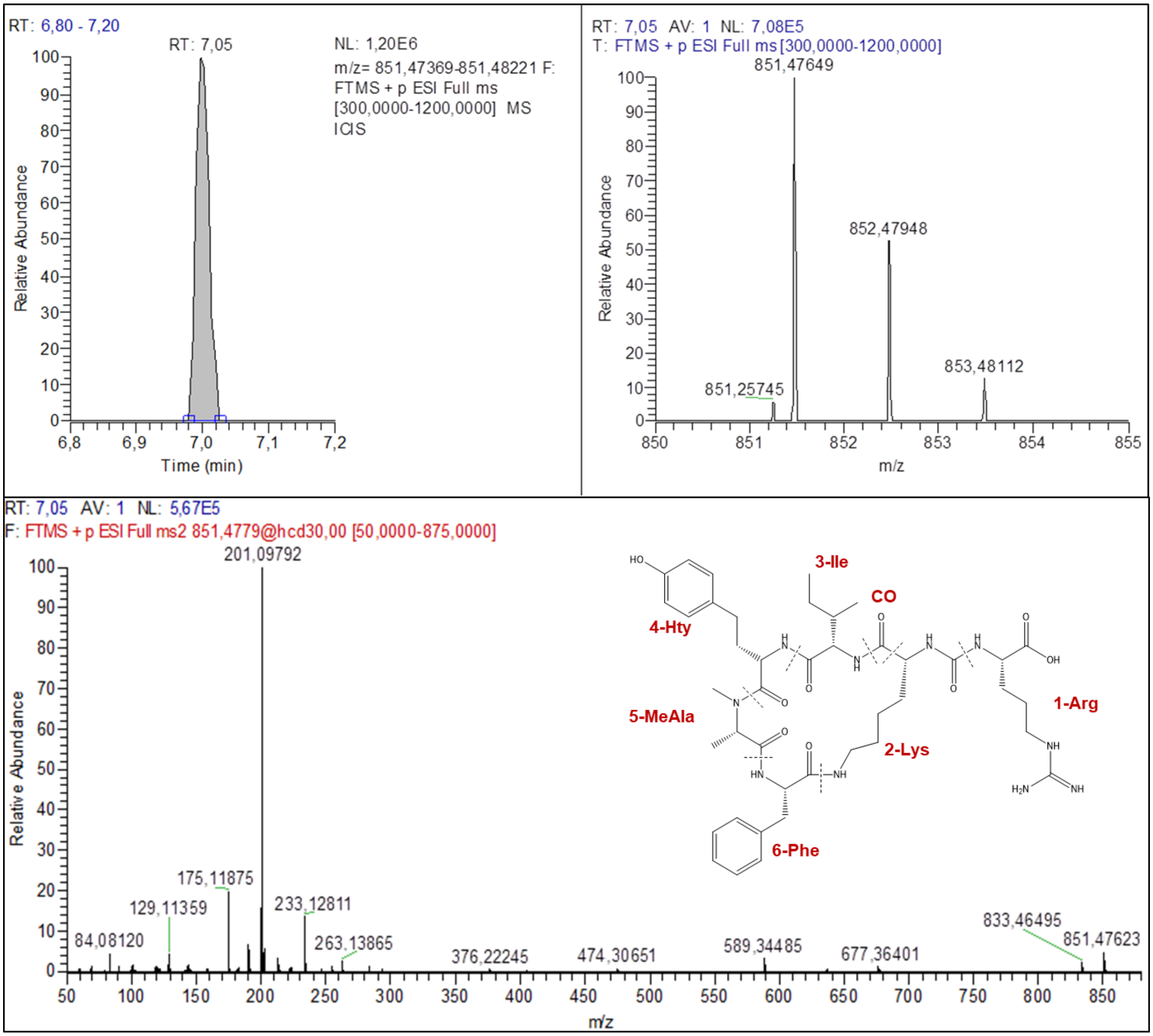

Figure S10. Chromatogram, isotopic pattern and fragmentation spectra of feature *m/z* 851.47649 identified as AP-F with RT at 7.05 min.

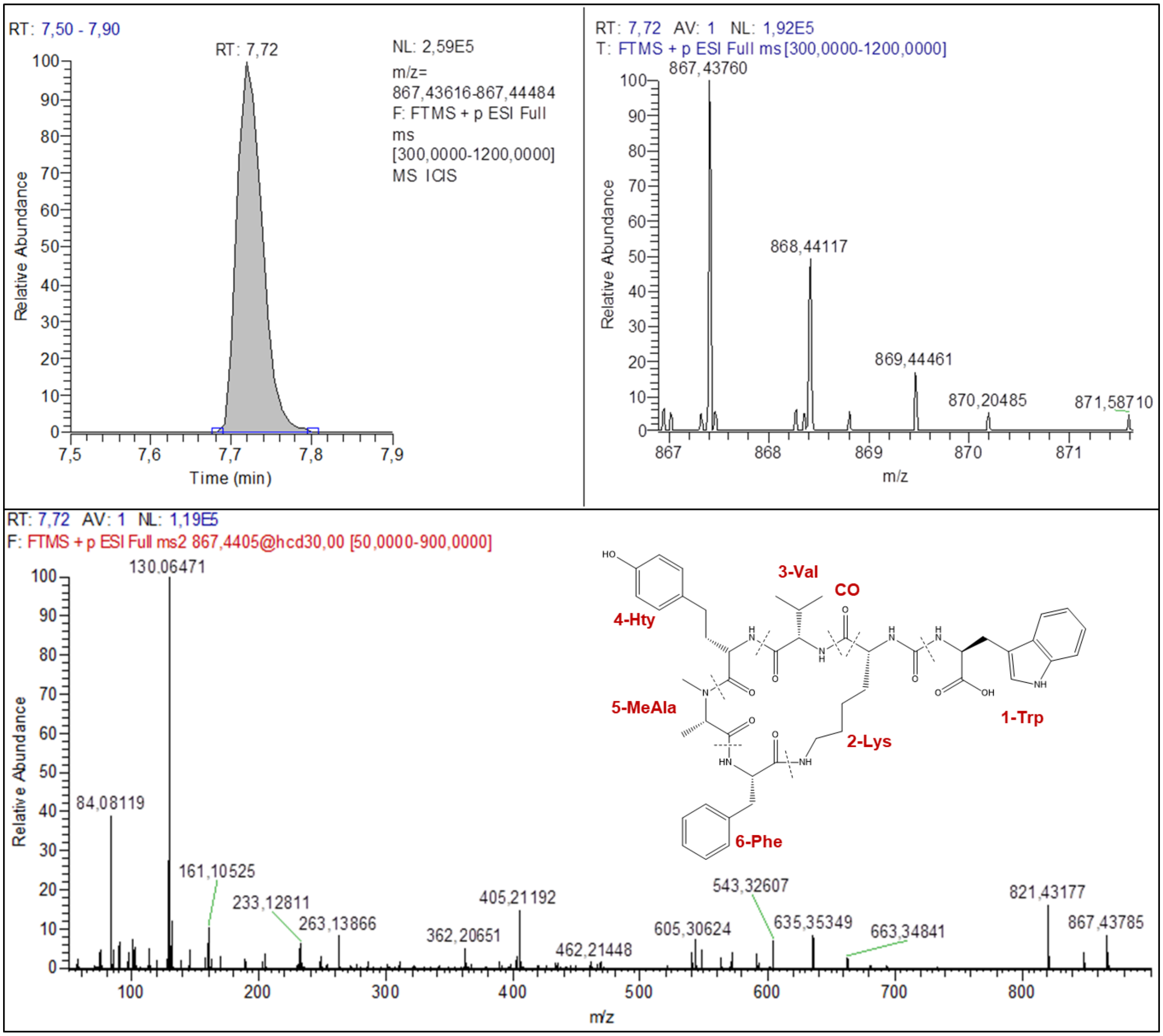

Figure S11. Chromatogram, isotopic pattern and fragmentation spectra of feature *m/z* 867.4376 identified as ferintoic acid A with RT at 7.72 min.

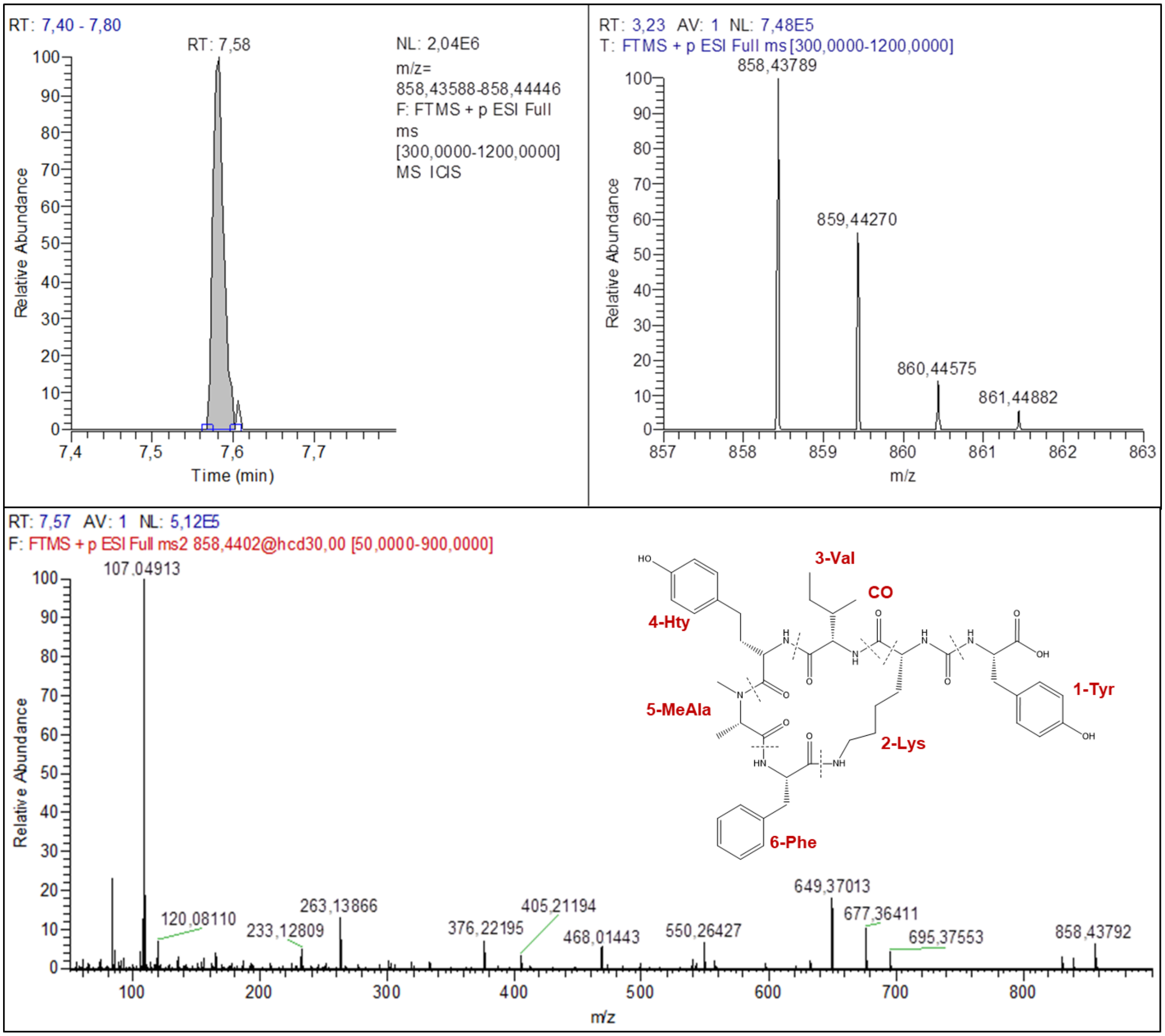

Figure S12. Chromatogram, isotopic pattern and fragmentation spectra of feature *m*/*z* 858.43789 identified as oscillamide Y with RT at 7.58 min.

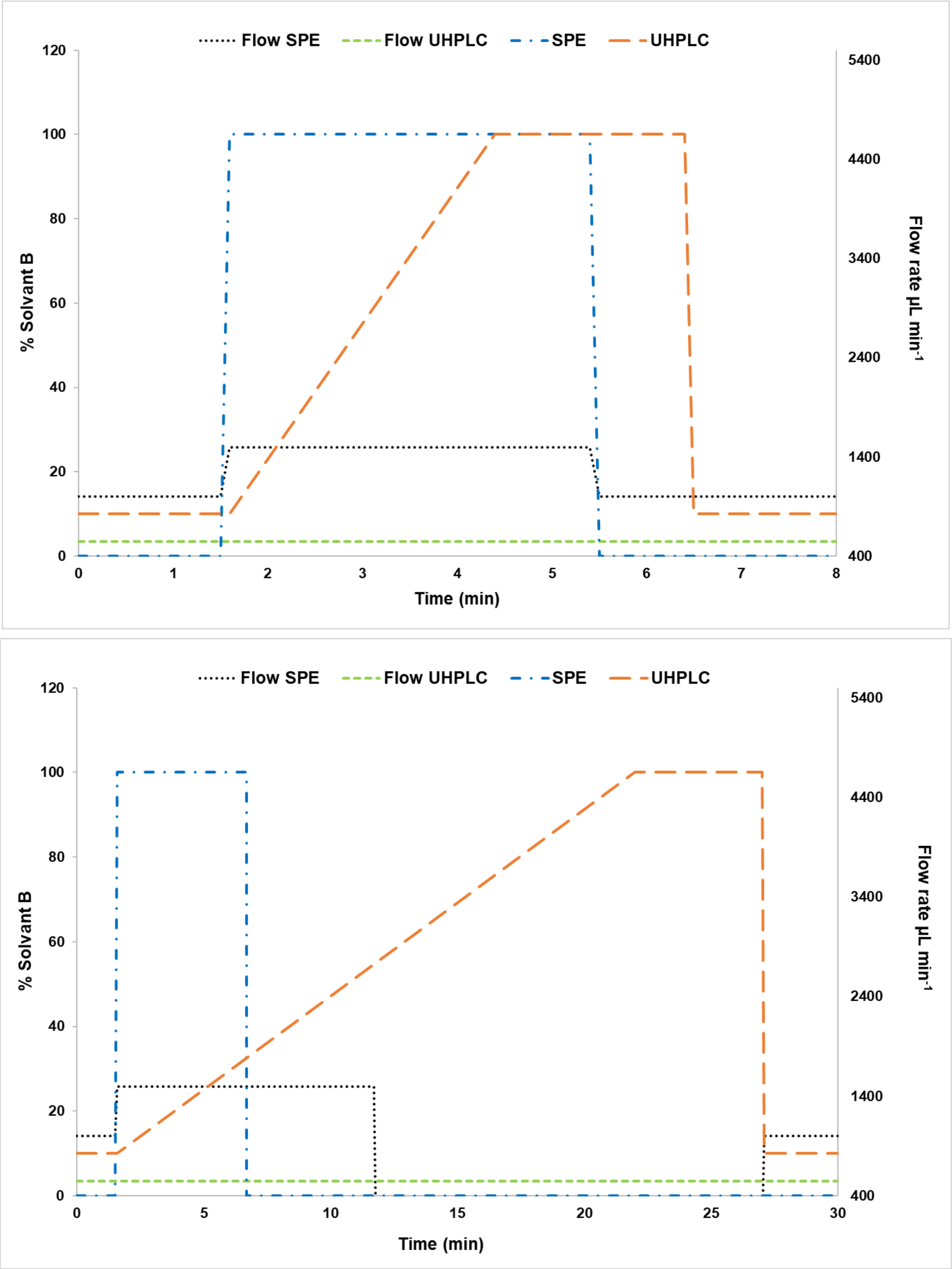

(a)

(b)

Figure S13. Details on the on-line SPE—UHPLC chromatographic gradient program for quantification (**a**) and suspect screening methods (**b**).

Table S5. Ionization and HRMS acquisition parameters.

**UHPLC/Ionization.**		**Target Analysis (Quantification)**			**Suspect Screening (Identification and Semi-Quantification)**		
Spray voltage	+3500 V		Full Scan	PRM	DIA (Full Scan)	DIA (MS/MS)	PRM
Capillary temperature	350 °C	Scan range (*m*/*z*)	150–1200	Inclusion list	300-1400	300–1400 divided in 22 windows	Inclusion list
Vaporizer temperature	250 °C	Scan time (min)	3.0-7.0	CYN: 1.5–3.0<br>ANA-a: 1.5–3.0<br>HANA-a: 2.0–3.1<br>MC-LA: 3.7–4.5<br>MC-LY: 3.7–4.7	1-27	1-27	4-15 (depending of the selected feature)
Sheath gaz flow	60 arbitrary unit						
Auxiliary gaz flow	15 arbitrary unit						
		Resolving power (FWHM *m*/*z* 200)	70,000	17,500	35,000	17,500	17,500
		AGC target (ions)	1×10^6	1×10^5	1×10^6	1×10^5	1×10^5
		Max injection time (ms)	200	50	100	50	50
